# In vivo grafting of large engineered heart tissue patches for cardiac repair

**DOI:** 10.1172/jci.insight.144068

**Published:** 2021-08-09

**Authors:** Richard J. Jabbour, Thomas J. Owen, Pragati Pandey, Marina Reinsch, Brian Wang, Oisín King, Liam Steven Couch, Dafni Pantou, David S. Pitcher, Rasheda A. Chowdhury, Fotios G. Pitoulis, Balvinder S. Handa, Worrapong Kit-Anan, Filippo Perbellini, Rachel C. Myles, Daniel J. Stuckey, Michael Dunne, Mayooran Shanmuganathan, Nicholas S. Peters, Fu Siong Ng, Florian Weinberger, Cesare M. Terracciano, Godfrey L. Smith, Thomas Eschenhagen, Sian E. Harding

**Affiliations:** 1 National Heart & Lung Institute, Imperial College London, London, United Kingdom.; 2 Department of Cardiovascular Science, Hamburg University, Hamburg, Germany.; 3 Institute of Molecular and Translational Therapeutic Strategies, Hannover Medical School, Hannover, Germany.; 4 Department of Cardiovascular Science, University of Glasgow, Glasgow, Scotland, United Kingdom.; 5 Centre for Advanced Biomedical Imaging, University College London, London, United Kingdom.

**Keywords:** Cardiology, Stem cells, Arrhythmias, Cardiovascular disease, Human stem cells

## Abstract

Engineered heart tissue (EHT) strategies, by combining cells within a hydrogel matrix, may be a novel therapy for heart failure. EHTs restore cardiac function in rodent injury models, but more data are needed in clinically relevant settings. Accordingly, an upscaled EHT patch (2.5 cm *×* 1.5 cm *×* 1.5 mm) consisting of up to 20 million human induced pluripotent stem cell–derived cardiomyocytes (hPSC-CMs) embedded in a fibrin-based hydrogel was developed. A rabbit myocardial infarction model was then established to test for feasibility and efficacy. Our data showed that hPSC-CMs in EHTs became more aligned over 28 days and had improved contraction kinetics and faster calcium transients. Blinded echocardiographic analysis revealed a significant improvement in function in infarcted hearts that received EHTs, along with reduction in infarct scar size by 35%. Vascularization from the host to the patch was observed at week 1 and stable to week 4, but electrical coupling between patch and host heart was not observed. In vivo telemetry recordings and ex vivo arrhythmia provocation protocols showed that the patch was not pro-arrhythmic. In summary, EHTs improved function and reduced scar size without causing arrhythmia, which may be due to the lack of electrical coupling between patch and host heart.

## Graphical abstract

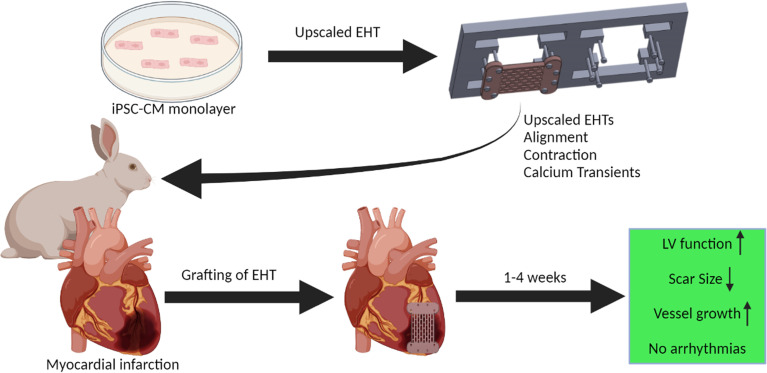

## Introduction

Heart failure is an emerging epidemic of ever-increasing incidence as the population ages, with severe morbidity and a 5-year mortality rate of 50% (1). Current treatments predominantly retard disease progression rather than actively reverse the disease process: therefore, there is an unmet need for novel therapies. The results of clinical trials in cardiac cell therapy, however, have been disappointing, with marginal benefits reported so far (2). This may in part be due to the existing delivery methods used in those clinical trials, with high degrees of washout and cell death within 24 hours postgrafting (3). Engineered heart tissue (EHT) is 3D reconstituted heart tissue containing human induced pluripotent stem cell–derived cardiomyocytes (hPSC-CMs), recapitulating many aspects of native myocardium (4), and may overcome the limitations of existing delivery methods (5). EHTs are effective in small animal models of heart disease, with improvements in cellular retention, vascularization, scar size, and heart function without the development of pathological ventricular arrhythmia (6, 7). These results show promise; however, these studies were predominantly conducted in small animal models with heart physiology dissimilar to humans and EHTs of an inappropriate size (10 *×* 1 mm with 1–7 *×* 10^6^ cells) (6). Direct myocardial injection of cardiomyocytes induced arrhythmia in pigs and nonhuman primate (NHP) models (8, 9). While scale-up to NHPs (or other large animal models, such as dog or pig) is essential for a tissue engineering therapeutic strategy, the cost and ethical problems prevent detailed hypothesis testing and improvement of the method.

As a new tool for this purpose, we have used the rabbit to produce an intermediate-sized model of myocardial infarction (MI) for EHT grafting (10–12). The rabbit heart is 50–100 times larger than mouse, but only 3 times smaller than macaque, which is the common NHP model. Compared with rodent, rabbit myocardium has closer similarities to human, including dominant β-myosin heavy chain expression, 70% of calcium cycling through the sarcoplasmic reticulum and a positive force frequency relationship, and a similar action potential profile and sarcolemmal ion channel expression (10–12). Unlike rodent, the morphology and ionic composition of the rabbit action potential is similar to the human. The anatomy of the circulation allows production of a reproducible infarct with similar epicardial to endocardial gradient as in human and comparable islands of cardiomyocyte survival throughout the scar (10, 13). Furthermore, immunosuppression protocols have been well studied in the rabbit, which is essential for xenograft studies (8, 9). Other important pragmatic considerations are the costs, which are 1–2 orders of magnitude lower than for pigs or NHPs, and the less acute level of ethical concern.

Here for the first time to our knowledge, we characterize upscaled hPSC-CM EHTs in a rabbit model of myocardial injury. In vitro, EHTs (25 *×* 15 mm) consisting of approximately 20 *×* 10^6^ cells were characterized for maturation over time before implantation. In the in vivo rabbit model, retention and host vascularization of EHTs were assessed over 4 weeks, as well as electrical coupling and arrhythmia burden. Overall, we demonstrate a positive effect on contraction of the infarcted heart without evidence for arrhythmogenic effects.

## Results

### EHT patches mature over time in vitro.

**Figure 1:**
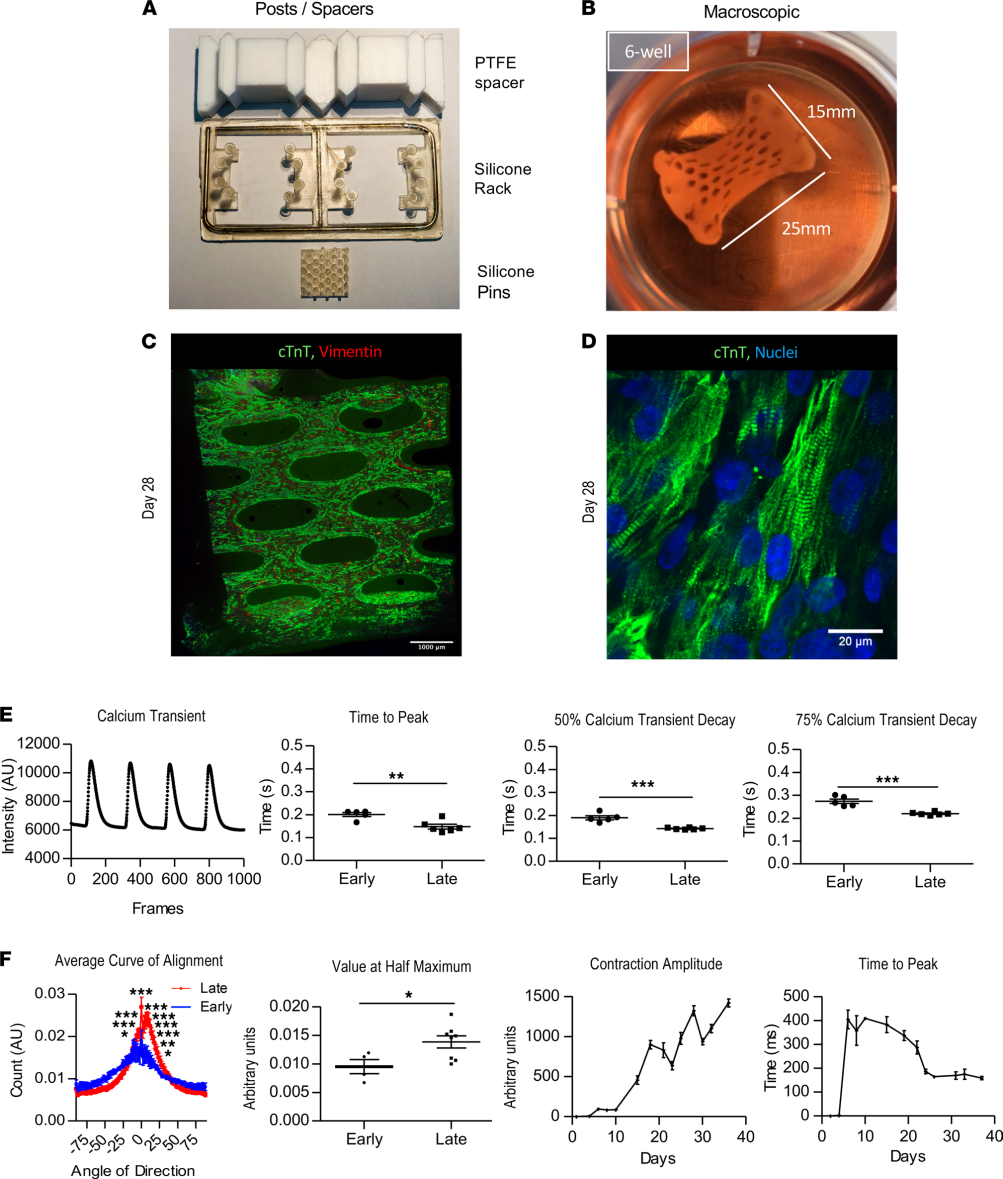
In vitro characterization of EHTs. (**A**) Equipment used to make silicone mold and to generate EHTs. (**B**) Macroscopic image of EHT just prior to grafting. (**C**) Immunostained EHT (troponin T abbreviated as TNNT2; fibroblasts; vimentin) 28 days after generation. Scale bar: 1000 μm. (**D**) Higher magnification image of **C** (TNNT2, fibroblasts, vimentin). Scale bar: 20 μm. (**E**) Graph of late EHT calcium transient (paced 1 Hz) and calcium transient differences between early and late EHTs (early, <2 weeks; late, >4 weeks). (**F**) Troponin T cell alignment data (*n* = 4 early; *n* = 8 late) and contraction kinetics over time (*n* = 6). Data are presented as mean ± SEM, and 2-way ANOVA and unpaired Student’s *t* test were the statistical methods used. *P < 0.05, **P < 0.01, and ***P < 0.001.****

Large EHT patches (25 *×* 15 *×* 1.5 mm) were created in vitro using upscaled silicone posts and Teflon spacers and matured in vitro for 3–4 weeks (Figure 1, A and B). At 4 weeks, troponin T–positive cells were homogenously distributed throughout the EHT (Figure 1C), and higher magnification images revealed well organized sarcomeres (Figure 1D). Calcium transients were then compared between recordings taken from early (4–14 days) and late EHTs (29–42 days). More mature calcium handling characteristics were obtained from late EHTs (Figure 1E), including faster time to peak measurements, faster 50% calcium transient decay measurements, and faster 75% calcium transient decay times. Troponin T–positive cardiomyocytes in later EHTs were also more aligned along the main force axis (Figure 1F), which was accompanied by improvements in contraction amplitude and time to peak contraction (Figure 1F).

### EHT cardiomyocyte retention drops over time but vascularization is consistent.

**Figure 2:**
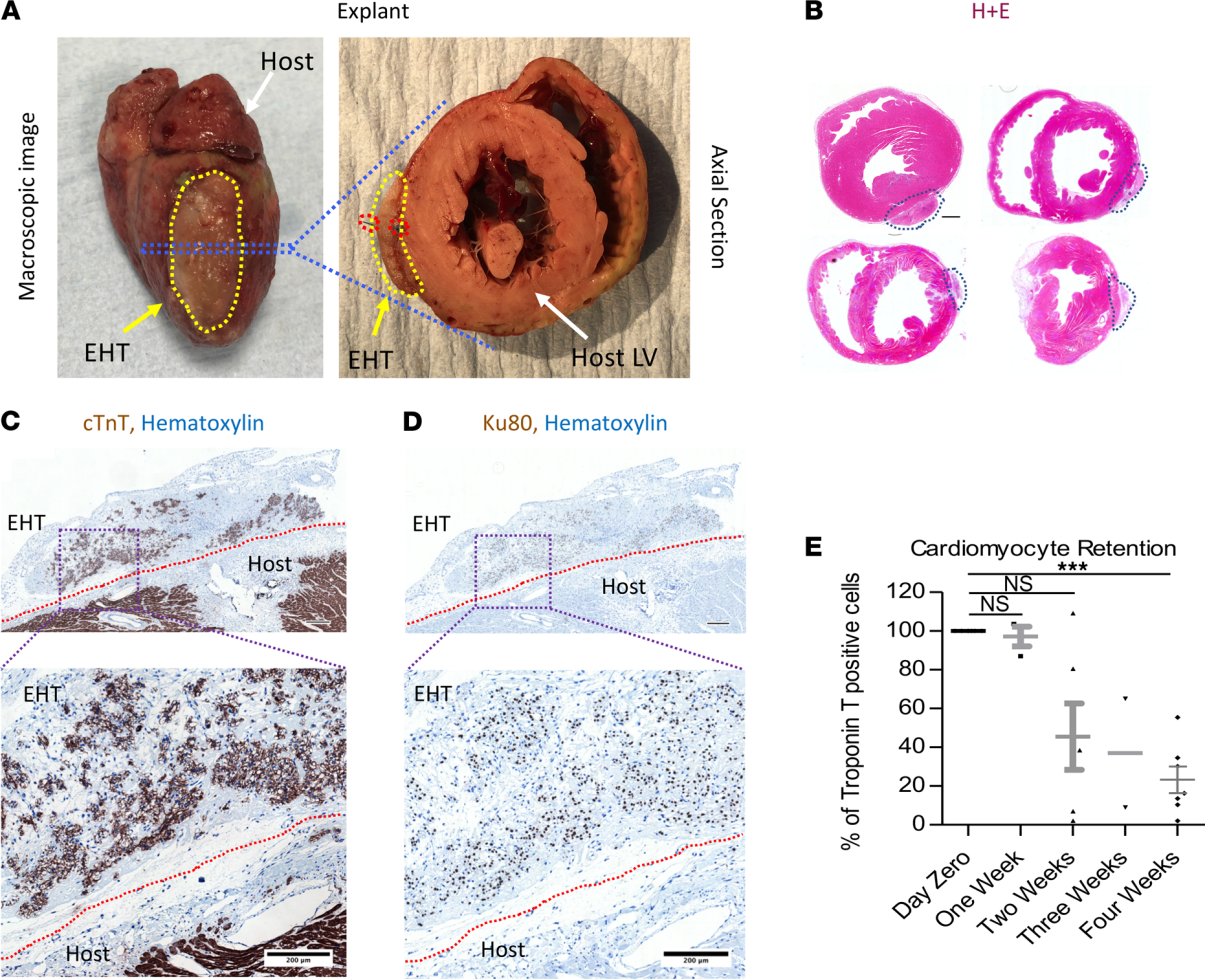
EHT grafting on controls. (**A**) Macroscopic image of EHT attached to epicardial surface 2 weeks postexplant. Red dotted lines show the EHT and host border zone. (**B**) Axial sections from 4 different animals postexplant (EHT outlined by blue dotted lines); scale bar: 1500 μm. (**C**) Troponin T (TNNT2) and hematoxylin staining of host/EHT; scale bar upper panel: 300 μm. (**D**) Ku80 and hematoxylin staining of host/EHT; scale bar upper panel: 300 μm. (**E**) Troponin retention at stratified time points relative to day 0. Data presented as mean ± SEM and 1-way ANOVA used to compare groups. ***P < 0.001.

Feasibility studies of transplantation of human upscaled EHT onto rabbit hearts are potentially novel, and therefore the first in vivo experiments were performed on control animals, without MI, at stratified time points up to 1 month (Figure 2). After explantation, large grafts could be visualized on the epicardial surface of the heart (Figure 2, A and B). Troponin T–positive cells in the grafts showed that cardiomyocytes were present in the graft (Figure 2C), and the presence of Ku80^+^ nuclei confirmed that the cells were of human origin (Figure 2D). There was no necrotic core in the EHT; however, there was a relative decrease of troponin T–positive cells farther away from the rabbit heart border (Supplemental Figure 1; supplemental material available online with this article; https://doi.org/10.1172/jci.insight.144068DS1). Retention was not significantly changed from day 0 (100%) to 1 week (97.1% ± 5.1%, *n* = 3, *P* = NS); however, at 2 weeks, the fraction of troponin T–positive cells was reduced (45.5% ± 17.1%, *P* < 0.05, *n* = 6), with a further reduction from 2 to 4 weeks (4 weeks 23.3% ± 6.8%, *n* = 7, *P* < 0.001; Figure 2E). A similar drop was seen in Ku80^+^ nuclei (Supplemental Figure 2), showing that this was hPSC-CM loss rather than decline in troponin per cell. The proportion of fibroblasts in the EHT (<5%) was consistent with the purity of the hPSC-CM differentiation (95%–98% TNNT2^+^ cells). It did not vary systematically over time in the patch in vivo, with the FSP1^+^ cell population remaining less than 10% from day 0 to week 4 (Supplemental Figure 3).

Interestingly, we found a significant number of cardiomyocytes proliferating, within the graft: 14.4% at 4 weeks compared with 2.46% at day 0 (Supplemental Figure 4, *P* < 0.05). The cardiomyocytes in the graft were less organized compared with native myocardium because there was an immediate loss of hPSC-CM alignment in the grafts on attachment to the heart when compared with in vitro on silicone posts (Supplemental Figure 5).

**Figure 3:**
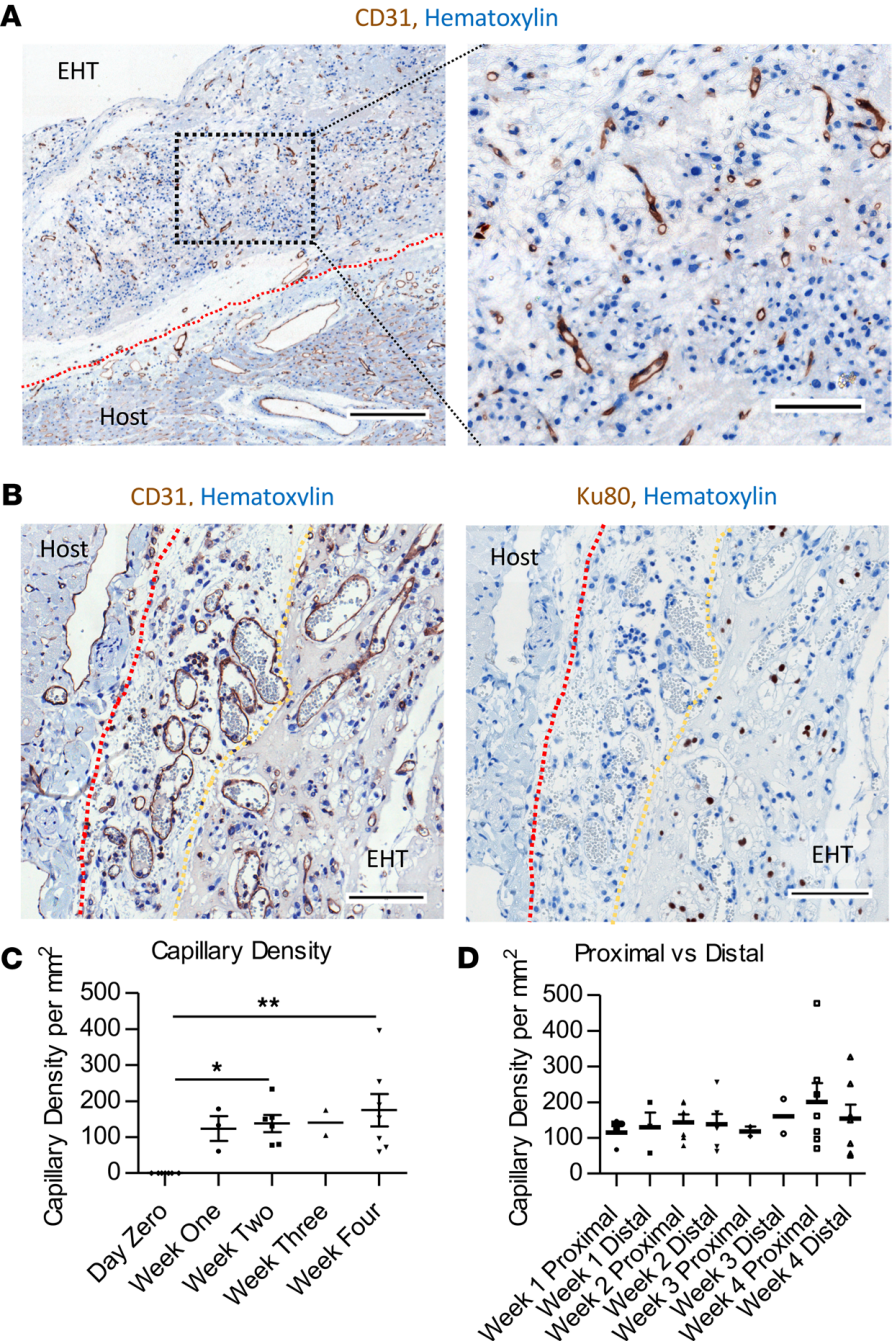
EHTs are vascularized by the host. (**A**) CD31 staining of EHT/host with higher magnification image (right panel); scale bar left panel: 300 μm; right panel: 100 μm. (**B**) CD31 (left panel) vessels and Ku80 nuclei stain of serial section; scale bar: 100 μm; red dotted line shows the heart; yellow dotted line shows the EHT border. (**C**) Graph of EHT capillary density over time. (**D**) Bar chart of capillary density of proximal and distal parts of EHT at serial time points (week 1 *n* = 3, week 2 *n* = 6, week 3 *n* = 2, and week 4 *n* = 7). Data presented as mean ± SEM and 1-way ANOVA used to compare groups. *P < 0.05, **P < 0.01.

Few CD31^+^ endothelial cells were present in EHT before implantation, but CD31^+^ vessels were present throughout the EHT from 1 week (Figure 3A): these were Ku80^–^, hence originating from the rabbit host myocardium (Figure 3B). Red blood cells could be observed in the lumen, indicating that flow had been established (Figure 3B). Capillary density increased from 0 before implantation to 123.9 ± 34.3/mm^2^ at week 1 and was consistent up to 4 weeks (Figure 3C). There was no significant difference between zones close to or distal from the rabbit heart border in capillary density at any time point up to 1 month postgrafting (Figure 3D).

In infarcted hearts, the percentage of cardiomyocytes in the grafts was reduced considerably: the percentage of troponin T–positive cells was 3.1% at 4 weeks, significantly decreased compared with day 0 (*n* = 3; Supplemental Figure 6) or with control hearts (*P* < 0.05). This was mirrored by a relatively low capillary density (30.1 ± 9.1/mm^2^, *n* = 3, *P* < 0.0015 relative to day 0; Supplemental Figure 7). The distribution of EHT cardiomyocytes in MI grafts did not show preferential localization proximal or distal to the host heart, although low numbers may have influenced our findings (Supplemental Figure 1).

### Immune cell infiltration in EHTs is related to MI rather than grafting per se.

Supplemental Figure 8 shows the presence of cells within the EHT showing the general hematopoietic marker CD45 or the specific macrophage marker Iba1. In EHTs that were implanted onto noninfarcted control hearts, there were few additional cells with these markers compared with day 0 (*P* > 0.05) at weeks 1–4 after implantation. In contrast, there were significant increases in infiltrating cells in EHT from MI hearts (*P* < 0.001 by ANOVA, weeks 1–4 vs. cell-free patch). This suggests that the presence of immune cells is more related to the inflammatory milieu of the infarcted hearts and that immunosuppression was sufficient to control the xeno-response to the hPSC-CMs within the patch.

### EHTs improve ventricular function when grafted onto infarcted hearts.

**Figure 4:**
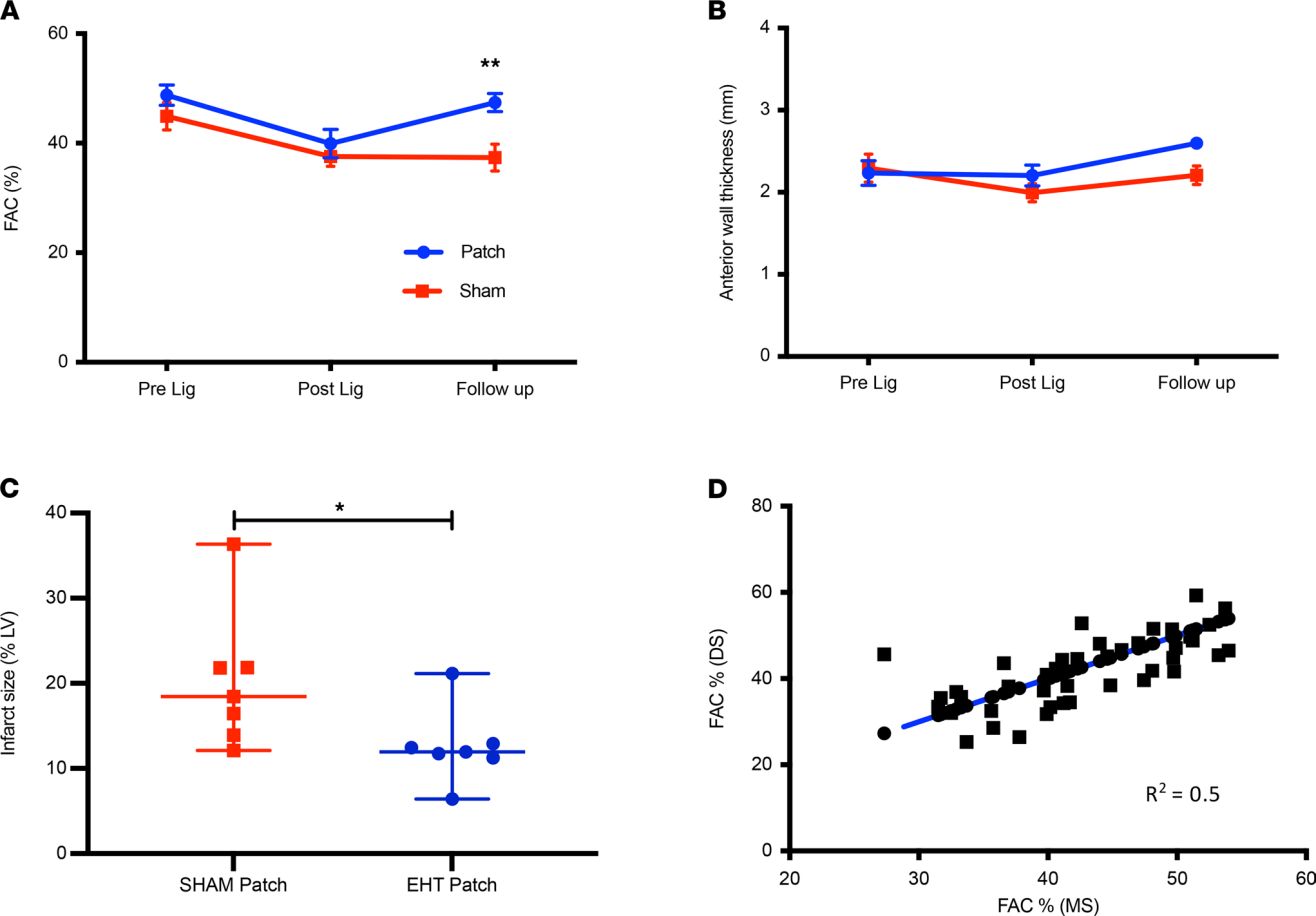
EHTs improved ventricular function when grafted onto infarcted hearts. (**A**) Fractional area change (FAC). **P < 0.01. (**B**) Anterior wall thickness (2-way ANOVA). (**C**) Quantification of Sirius red infarct staining of rabbit heart sections (*n* = 7 sham; *n* = 7 EHT: sham: 1 week *n* = 1; 4 weeks *n* = 6; EHT 1 week *n* = 1; 2 weeks *n* = 2; 4 weeks *n* = 4). *P < 0.02, Mann-Whitney *t* test. (**D**) Correlation of FAC measurements between 2 echocardiographers blinded to treatment allocation (*n* = 42 measurements). Data presented as mean ± SEM (**A** and **B**); data presented as median with 95% confidence intervals (**C**).

Efficacy was tested by an experienced blinded echocardiographer at time points up to 1 month, and a significant improvement was seen in the EHT-treated infarction group (*n* = 7; fractional area change = 10.04% ± 3.1%, *P* < 0.01, Figure 4A). This was associated with a reduction in infarct scar size in the EHT group (11.9 [95% CI 6.4%–21.1%] [patch] vs. 18.4 [95% CI 12.2%–36.3%] [sham], *P* < 0.02) (Figure 4C). There was no significant difference in anterior wall thickness between sham and EHT groups at explant (Figure 4B). There were no significant differences in left atrial diameter (12.9 ± 1.0 mm [patch] vs. 11.6 ± 0.9 mm [sham]; *P* = NS), left ventricular end diastolic diameter (17.7 ± 0.6 mm [patch] vs. 16.6 ± 0.8 mm [sham], *P* = NS), and left ventricular end systolic diameter (11.8 ± 0.4 mm [patch] vs. 11.7 ± 0.7 mm [sham], *P* = NS) between EHT and sham controls at the time of explant. To ascertain the reliability of measurements, a second experienced blinded preclinical imaging expert assessed echocardiography images with a good correlation between measurements, therefore confirming the validity of our analysis (Figure 4D).

### Optical mapping of explanted hearts.

**Figure 5:**
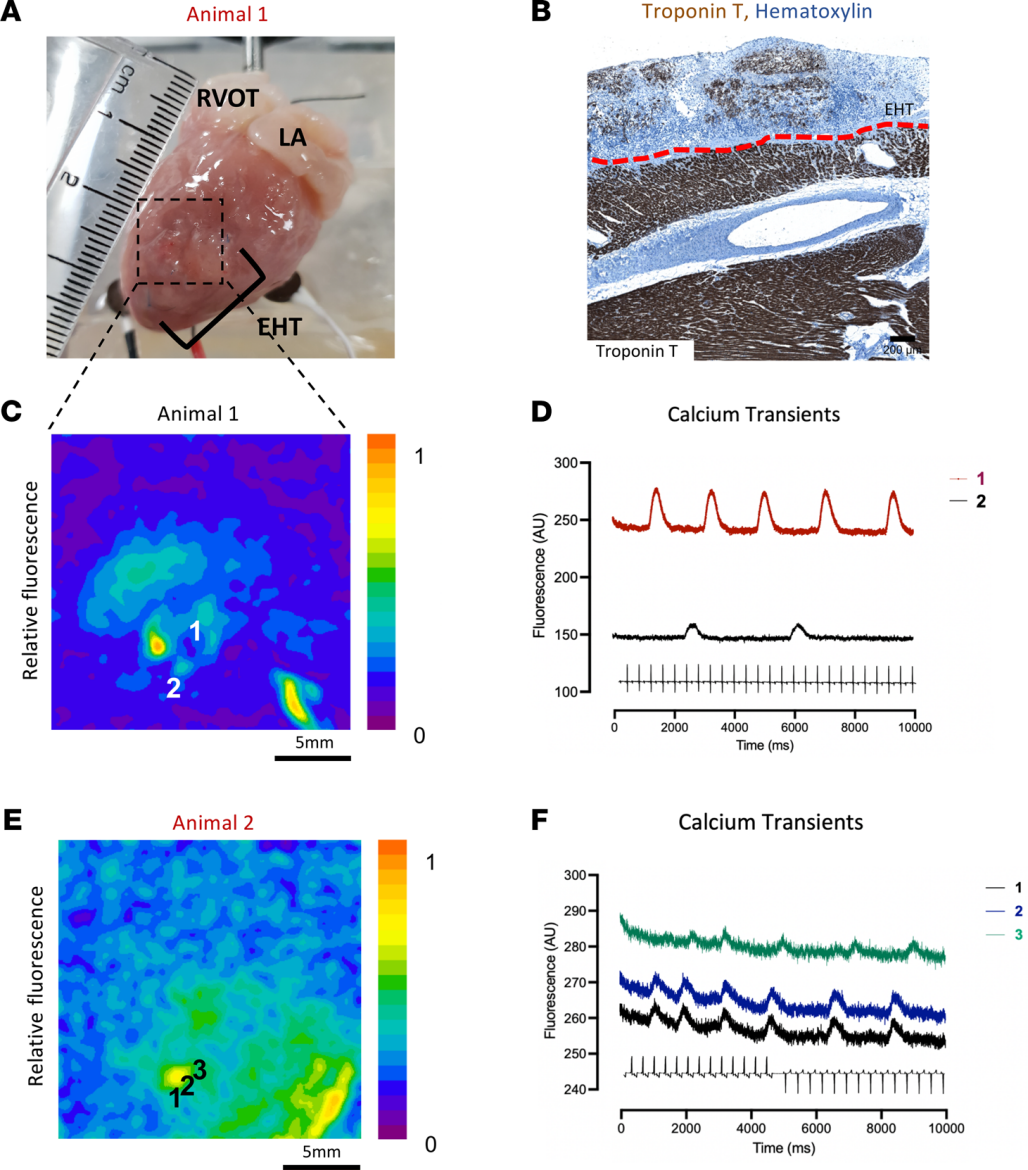
EHT optical mapping. (**A**) Macroscopic image of EHT/rabbit heart explanted and mapping 1 week postgrafting. (**B**) Troponin T staining (brown area) of animal in **A**, host/EHT separated by red dotted line; scale bar: 200 μm. (**C**) Fluorescence map of animal in **A**; (**D**) calcium transients of **C**; numbers correspond to numbers in **C**. (**E**) Fluorescence map of animal 2 weeks postgrafting. (**F**) Respective calcium transients of **E**.

Electrical coupling of the grafted EHT to the host is one of the critical questions regarding EHT as modality of treatment in the regenerative medicine field (as opposed to intramyocardial injection). In total, 9 control (*n* = 1 at week 1; *n* = 3 at week 2; *n* = 1 at week 3; *n* = 4 at week 4) animals underwent ex vivo optical mapping studies (representative images in Figure 5, A and B). In 4 out of 9 hearts, there was evidence of spontaneous calcium transient activity (GCaMP6f fluorescence derived from hPSC-CMs) found in the graft. Fluorescence maps and calcium transients from the EHT are shown in Figure 5, C–F. The spontaneous rates were slower than the intrinsic rate of the host (week 1: 24 bpm; week 2: 26 bpm; week 3: 27 bpm; week 4: 78 bpm; versus approximately 220–300 bpm in host), which argues against coupling of the graft. There was no conclusive evidence of electrical coupling between host and EHT during sinus rhythm, ventricular pacing, or EHT pacing.

### EHTs are not arrhythmogenic in nature.

**Figure 6:**
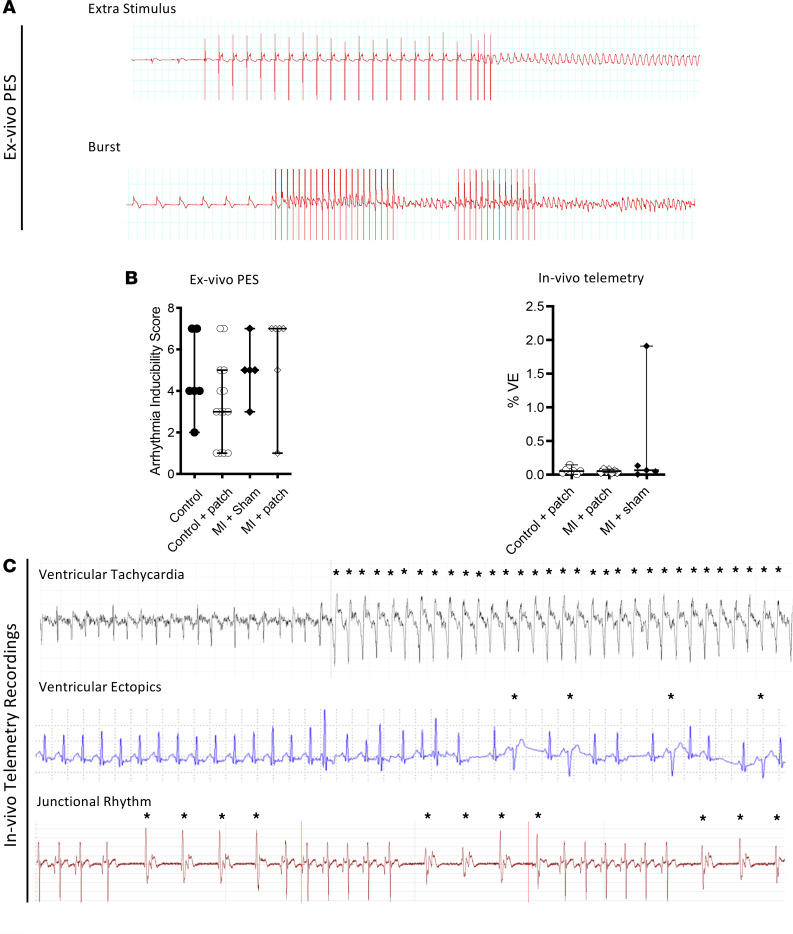
Arrhythmogenic potential of EHTs. (**A**) Representative ECG traces obtained from ex vivo programmed electrical stimulation protocols. (**B**) Arrhythmia inducibility scores (ex vivo) and in vivo ventricular ectopics (VE) burden (%) recordings obtained from in vivo telemetry. (**C**) Representative traces of ventricular tachycardia, VE, and junctional rhythm obtained from in vivo telemetry recordings. Data presented as median and 95% CI and Kruskal-Wallis test used to compare groups. *P < 0.05.

Direct myocardial injection of cardiomyocytes induces arrhythmia in pigs and NHP models (8, 9). The arrhythmogenic potential of EHTs here was assessed ex vivo and in vivo by programmed electrical stimulation (using burst or extra stimulus) and intermittent telemetry recordings, respectively (Figure 6). Ex vivo programmed electrical stimulation protocols revealed that there was no difference in arrhythmia thresholds between infarcted hearts that underwent sham patch implantation and EHT implantation (Figure 6B). In 1 animal (from 7) in the EHT/MI group, multiple runs of unsustained ventricular tachycardia were found in a single 24-hour period of recording (day 15 from a total of 28 days) lasting 59 beats; 17 beats; 50 beats and 17 beats (Figure 6C). Clinically relevant sustained arrhythmias (e.g., >30 seconds’ duration of ventricular tachycardia or ventricular fibrillation) were not detected in any control animals. In the MI/sham patch group, sustained ventricular tachycardia was detected in 1 animal (day 11): however, this too survived until the 4-week point. In all groups, the overall incidence of ventricular ectopic beats was low.

## Discussion

The main findings of this study are a) grafting of upscaled EHTs is feasible and safe in a rabbit model of MI; b) EHTs are rapidly vascularized throughout; c) EHT grafting improved function and reduced infarct size when grafted onto infarcted hearts; d) while the grafted EHTs displayed active calcium transients, no coupling to host heart could be detected; and e) EHTs were not proarrhythmic in nature.

When compared with contemporary cell injection modalities (intracoronary/intramyocardial injection), tissue engineering strategies offer several potential benefits, including a) vascularization with prolonged cell survival and paracrine release, b) the ability to provide structural support to the failing ventricle, and c) the ability to cover scarred tissue with engineered heart muscle with potential to provide contractile improvement (14). The field has undergone a steady evolution since the original constructs using collagen and neonatal rat cardiomyocytes, but there are still a number of potential barriers that need addressing prior to clinical translation, including upscaling, testing in more relevant cardiac models, and understanding electromechanical integration (4, 6).

In this study, we aimed to upscale EHT to target the scar in infarcted rabbit heart. Patches of this size can potentially contain up to 50 million hPSC-CMs each, and therefore 10 patches deployed in a human heart would deliver half a billion cardiomyocytes, a clinically relevant number. While a single large patch would be desirable, multiple patches of the present size could alternatively give flexibility in surgical deployment around the scar border. Patches were matured in vitro using dynamic rocking culture to increase nutrient availability compared with static cultures and by using flexible silicone posts to provide auxotonic load (15). Patches matured over time in vitro and displayed improvements in cardiomyocyte alignment, calcium transients, and contraction kinetics after 3–4 weeks in culture. Other groups have reported larger EHTs but have not reported such extensive in vivo preclinical experiments (5, 14, 16, 17).

The in vivo study of patches was assessed for the first time to our knowledge in a rabbit model, which has specific advantages because of the numerous similarities to human myocardium, including a positive force frequency relationship and similar excitation-contraction coupling (18). Compared with smaller model organisms the rabbit has a more similar heart rate, systolic pressure, and contractility to humans (Table 1). Using permanent ligation (rather than ischemia/reperfusion) results in a rabbit postmyocardial infarction syndrome that has human parallels, including inversion of the force frequency relationship, desensitization of β-adrenoceptors, and residual transmural islands of cardiomyocytes (10, 19–21). Ideally, implantation will move toward the chronic heart failure phase of the model, to reproduce the likely clinical use of the patch. However, implantation of the EHT at a later stage brings added complexity because of the second thoracotomy, and so the present study to establish the feasibility of patch implantation is an important step.

Previous grafting studies that tried to move away from rats have used guinea pigs, pigs, and NHPs. Guinea pigs have multiple collateral coronary arteries, so if the left anterior descending artery and left circumflex artery are ligated, blood is still supplied normally to the ventricle (22). Cryoinjury is therefore used to create a damaged area, but this may not be exactly equivalent to an infarct. Pigs have similar cardiac physiology to humans in many ways; however, they have Purkinje fibers that almost transverse the whole of the myocardium (23). Therefore, in similar-sized hearts, standard electrophysical measurements, such as QRS and QT intervals, are much faster than in humans (24). Larger animals are important for clinical translation of tissue engineering applications, but the range of heart rates and left ventricular end diastolic diameters are overlapping between macaque and rabbit (Table 1). We suggest that the rabbit could be a more cost-effective and less ethically problematic model than the macaque, particularly for the hypothesis-testing phase of clinical development.

Establishing experiments were performed on controls, and efficacy was tested on an ischemic myocardial injury model. Grafts were extensively vascularized by the host: this was maximal within 1 week and maintained until week 4. The homogenous vascularization suggests that there may have been ingrowth of vessels from the pericardial cover as well as from the rabbit heart itself. It was noticeable that host vessels were present across an observed fibrous layer. However, the final capillary density was considerably lower than that in normal adult myocardium (>2000 capillaries/mm^2^) (25). Troponin T retention in controls was relatively sustained at 1 week, dropping to approximately 20% at week 4. Possible reasons for cell loss are immune attack of the xenograft, due to incomplete immunosuppression, or hypoxic hPSC-CM death. For the first point, neither general nor macrophage-specific markers were higher in noninfarcted control hearts than day 0, or sham, after patch grafting at 1, 2, or 3 weeks. There was a slight rise at week 4, but even this was considerably lower than the values in MI hearts. This suggests that immune activation by the patch itself is low and that the majority of infiltration is related to the infarction. For the second point, vascularization is clearly rapid, being established by week 1, and well distributed, but is lower than that in normal myocardium. There is therefore a potential for hypoxic damage, especially at later times when the protective effects of the prosurvival cocktail will have declined, and the hPSC-CMs in the patch may have matured (therefore becoming more oxygen reliant). This explanation could be consistent with the greater loss distal to the rabbit heart border, although a necrotic core would have been more likely if hypoxia were the cause.

Cellular retention in infarcted animals was substantially lower than in controls at week 4. We also saw 10-fold less vessel infiltration into the scar area in infarcted animals, so hypoxia could be a stronger factor in the cell death here. Likely, the greater inflammatory reaction that accompanied infarction could also have increased cell death within the graft. Another possibility is atrophic muscle loss due to decreased load. It was noticeable that sarcomeric alignment dropped immediately on grafting, probably because the tension on the patch was now less than on the flexible silicone posts (where passive tension increases as the patch condenses). Interestingly, we detected cardiomyocytes proliferating within our grafts, similar to previous reports (7). The loss of sarcomeric protein over the 4 weeks may have allowed the proliferative activity to rise. In future work, encouragement of controlled graft proliferation presents an opportunity to offset hPSC-CM loss over time.

The combination of in vivo grafting and ex vivo optical mapping is a powerful tool to investigate graft/host coupling in the rabbit. Electromechanical integration was assessed using a perfused membrane potential reporter to track excitation in the (chemically arrested) rabbit heart and calcium transient signals from GCaMP6f hPSC-CMs to monitor the EHT. Spontaneous calcium signals were observed in 4 out of 9 hearts, although the later loss of hPSC-CMs (or reporter silencing as previously reported) (7, 14) may have reduced the GCaMP6f signal. The lack of definitive electromechanical integration is consistent with the literature of patch therapy (7, 14) and may be due to the fibrous layer between the graft and the host that could prevent electrical coupling.

Based on the data presented so far in this paper, we are unable to provide information regarding the exact mechanism of the improvement in left ventricular function, but it is unlikely that this is entirely due to remuscularization given the low cell retention observed at 4 weeks. However, possible mechanisms of benefit could include the antiinflammatory and antiapoptotic paracrine factors that are secreted by the hPSC-CMs, especially since a reduction in infarct size was seen in the infarct group and lack of electrical coupling observed. There was also clearly an angiogenic signal that attracted the vessels from the rabbit heart into the EHT, and this could have been a more generalized vasculogenic response that benefited the host. In a study by Gao et al., similarly upscaled EHT patches (4 *×* 2 cm) containing hPSC-CMs (and noncardiomyocytes) were grafted onto a swine model of MI, and efficacy was shown with a relatively small number of hPSC-CMs (4 million). Since there was a 10% engraftment rate at 4 weeks, they suggested that paracrine mechanisms were the predominant mechanism of efficacy; however, there was no distinction made between paracrine- and immune-related actions (16).

Arrhythmia and therefore safety of EHT patches for clinical use was assessed using in vivo telemetry recordings and ex vivo using programmed electrical stimulation protocols. Importantly, no clinically relevant arrhythmias were observed in any animal that underwent EHT grafting. Furthermore, arrhythmia provocation protocols did not reveal any significant differences between the EHT and cell-free patch groups. These findings are key for the regenerative medicine field and in contrast to the intramyocardial cell delivery route in which pathological ventricular arrhythmia has been reported previously (3, 9, 16, 26). The lack of arrhythmia observed may be due to the lack of direct electrical coupling observed. This leaves an important question of whether and how the hPSC-CMs in the patch could contribute to mechanical force. A further potential mechanism of functional coupling is through mechanical stimulation of the EHT by the contraction and relaxation of the host heart: mechanical stimulation can be as effective as electrical in pacing the rabbit heart (27).

In summary, we were able to successfully upscale EHT to a clinically relevant size and test EHT in an intermediate-sized model of ischemic myocardial injury. The EHTs displayed evidence of maturity after 3–4 weeks in culture, and the experiments presented in this report are the first to our knowledge to evaluate the use of large EHT patches in a preclinical rabbit model with extensive similarities to human myocardium. EHT grafting was associated with improvements in left ventricular function and reductions in scar size and importantly was not associated with any significant development of arrhythmogenicity. EHTs may therefore be the preferred modality to use in delivering cell therapy to the failing heart.

## Methods

A detailed description of the methods can be found in the Supplemental Methods. Supplemental Videos 1-4 are examples of EHT patches beating at various time points.

### Study design

**Figure 7:**
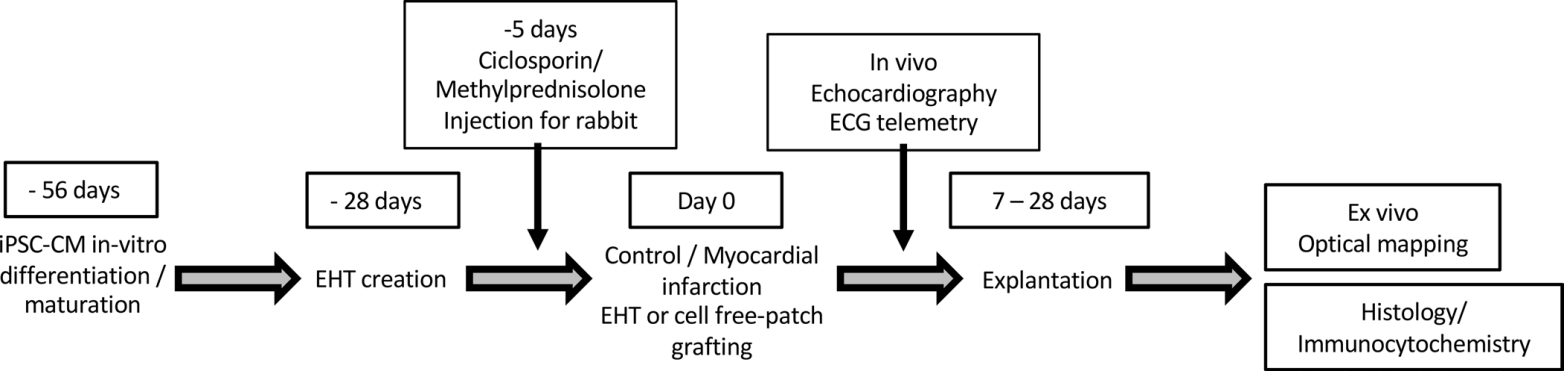
Timeline for in vivo experiments. In vivo grafting protocol.

The purpose of this study was to develop and characterize upscaled EHT in vitro and test: a) feasibility of grafting onto control rabbit hearts and b) efficacy in a myocardial injury model. Permanent ligation of the marginal artery was chosen to mimic MI because consistent transmural infarcts can be generated. Animals were assigned to treatment groups by an independent technician, and echocardiography was assessed by a group-blinded observer and check scored by a second independent blinded observer. Arrhythmia was assessed in vivo and ex vivo and histological examination carried out on all samples to assess vascularization, troponin T–positive cell retention, and tissue maturation at various time points. Ex vivo optical mapping was performed to assess graft calcium transient activity and degree of electrical coupling to the host heart. A timeline for the procedures and in vivo and ex vivo assessments is shown in Figure 7.

### Animal husbandry and induction of MI

New Zealand white male rabbits (2.5–3.5 kg) were obtained from Envigo Laboratories. MI was performed as previously described (10) and as in Supplemental Methods. EHT grafting was performed after artery ligation, and the EHT or sham (acellular) patch was placed over the expected infarct territory. Immunosuppression consisted of ciclosporin (10 mg/kg) 5 days prior to grafting and methylprednisolone (2 mg/kg) 48 hours before EHT implantation and continued until explantation. The protocols carried out conformed to The Animals (Scientific Procedures) Act 1986 Amendment Regulations 2012 and EU directive 2010/63/EU.

### Generation of EHTs and grafting

hPSC-CMs incorporating the calcium-sensitive reporter GCaMP6f were maintained in TeSR-E8 and differentiated as described previously (28). EHTs were upscaled to a 6-well size using the same protocol as the 24-well format (29). For these, 15 *×* 10^6^ to 20 *×* 10^6^ hPSC-CMs (95%–98% purity) were mixed with fibrinogen and thrombin to produce spontaneously and synchronously beating hydrogel constructs, which were fed daily until use at 4 weeks. Patches were matured in vitro using dynamic rocking culture to increase nutrient availability and using flexible silicone posts to provide auxotonic load (15). Grafts (both sham and cellular patch) were treated with a prosurvival cocktail and heat shock regimen starting 24 hours prior to grafting: this has been protective in other studies with hPSC-CMs (30). EHTs were secured to the epicardium of rabbit hearts with 7.0 Prolene sutures when the animal was stable postligation (25–30 minutes), and once secure, a pericardial cover was placed over the graft.

### Endpoints

#### Measurement of heart function.

Efficacy was tested by an experienced blinded echocardiographer at stratified time points at weekly intervals up to 1 month. Echocardiography was performed by an experienced sonographer on a clinical-grade IE33 machine (Phillips), and analysis was performed by 2 reviewers blinded to the treatment groups (each data point compared was an average of 3 separate recordings). Parasternal long axis and parasternal short axis views were taken to assess left atrial size, wall thickness, and FAC. FAC was calculated as (LVEDA – LVESA)/LVEDA; LVEDA = left ventricular end diastolic area; LVESA = left ventricular end systolic area (7).

#### Assessment of arrhythmia.

Telemetry recordings were carried out to assess the arrhythmogenicity of EHT grafts using 2 devices: Reveal Linq (Medtronic) and Small Animal Telemetry System (Indus Instruments). Full details are provided in the Supplemental Methods. Arrhythmia inducibility was assessed ex vivo by programmed electrical stimulation in Langendorff-perfused hearts (31). Full details are described in the Supplemental Methods.

#### Optical mapping.

Full details are described in the Supplemental Methods. Data were analyzed with QRecord software as previously described (32).

### In vitro characterization

#### Contractility.

EHT contraction was measured using MUSCLEMOTION software on a custom 3D printed apparatus (33).

#### Calcium transients.

GCaMP6f EHTs were analyzed using standard methods (see Supplemental Methods), and parameters were calculated in pClamp. GCaMP6f cells were obtained courtesy of the Conklin laboratory (Gladstone Institutes, San Francisco, California, USA).

### Statistics

The data from the experiments herein were analyzed in GraphPad Prism 9 software. For differences between 2 groups, an unpaired 2-tailed Student’s *t* test was used. Statistical differences in more than 2 groups were determined by 1-way ANOVA (parametric or nonparametric as applicable) with Tukey’s post hoc test. For mean differences of groups with 2 independent variables, 2-way ANOVA was used. Data are presented as mean with error bars representing SEM. A *P* < 0.05 was considered significant with *P < 0.05, **P < 0.01, and ***P < 0.001.

### Study approval

Every procedure was performed according to the standards for the care and use of animal species written in the *Guide for the Care and Use of Laboratory Animals* (NIH publication no. 85-23, revised 1996, National Academies Press, 2011). In addition, experiments conformed to the dedicated requirements as set by the UK home office (ASPA 1986; Amendments Regulations 2012), which incorporates the EU directive 2010/63/EU. All the protocols carried out had prior approval from the Animal Welfare and Ethical Review Body of Imperial College London, London, United Kingdom.

## Author contributions

RJJ and TJO contributed by designing research studies, conducting experiments, acquiring data, analyzing data, and writing the manuscript. RJJ created the rabbit model and performed most of the grafting experiments at Imperial College London and therefore is numerically first. PP, MR, BW, OK, LSC, DP, and DSP conducted experiments and acquired data. RAC, FGP, BSH, WKA, and FP conducted experiments, provided reagents, acquired data, and analyzed data. MD performed experiments and analyzed data. RCM, DJS, MS, and NSP analyzed data and revised the manuscript. FSN, FW, CMT, GLS, TE, and SEH designed research studies and wrote or critically revised the manuscript.

## Supplementary Material

Supplemental data

## Figures and Tables

**Table 1:**
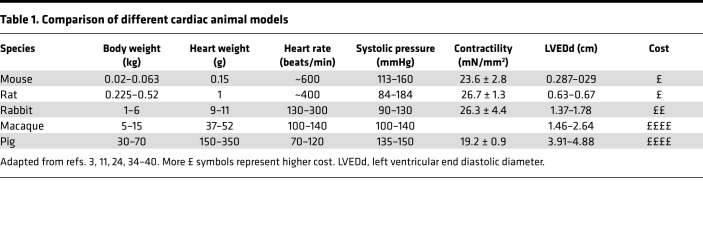
Comparison of different cardiac animal models

## Version 1 08/09/2021

Electronic publication
