# Supplementary material for: In vivo grafting of large engineered heart tissue patches for cardiac repair
Source: JCI Insight. 2021 Aug 9;6(15):e144068. doi: 10.1172/jci.insight.144068 (PMC8410032; doi:10.1172/jci.insight.144068)
Supplement: Supplemental data [file jciinsight-6-144068-s077.pdf]

Supplementary Information

This supplementary document includes:

- Methods
- Supplementary Figures 1-8
- Supplementary Videos 1-4

Methods

Animal husbandry and induction of MI

Male New Zealand white male rabbits (2.5-3.5kg) were obtained from Envigo Laboratories (Huntington, UK). Animals were anesthetised with ketamine (7.5mg/kg) and medetomidine hydrochloride (0.125mg/kg) and the operating area was shaved. Intubation was carried out using a V-gel airway (Docsinnovent) and animals were ventilated according to weight (35-50 breaths per minute, tidal volume: 10-15mls/kg). Then drugs were administered as follows:

**Drug**	**Administration**	**Dose**
Isoflurane	Ventilator	Maintenance (1-4%)
Rimadyl	Subcutaneous	4mg
Antisedan	Intramuscularly	0.125mg/kg
Domitor	Subcutaneous	0.125mg/kg
Baytril	Subcutaneous	10mg/kg
Marcaine (0.5%; 5 mg/mL)	2nd, 3rd, 4th, 5th, and 6th intercostal spaces	0.2ml each rib
Quinidine gluconate	Intravenous - Cannulated ear vein (bolus prior to artery ligation)	10mg-20mg depending on HR response

The fourth intercostal rib space was opened, and a small cut made in pericardium so the heart can be removed. This also preserves the pericardium so it could be used as a pericardial cover after the EHT has been grafted to the epicardial surface. A 4.0 Prolene suture was placed in the apex of the heart to provide manoeuvrability and

the marginal artery ligated with a 4.0 Prolene suture 5-7mm from atrioventricular border. Immunosupression consisted of ciclosporin (10mg/kg, oral) five days prior to grafting and methylprednisolone (2mg/kg, intramuscular) 48 hours before EHT implantation and continued until explantation. The protocols carried out had prior ethical approval by Imperial College London and conformed to The Animals (Scientific Procedures) Act 1986 Amendment Regulations 2012, and EU directive 2010/63/EU.

Cell maintenance and differentiation

Undifferentiated stem cells (IMR-90, gCaMP6F [provided courtesy of Conklin Laboratory, Gladstone]) were maintained in culture with mTeSR/TeSR-E8 (Stem Cell Technologies) and were then split (1:15) ratio by using versene (Thermo Fisher Scientific). Seeding was carried out with 10µM Y-27632 (Sigma-Aldrich) onto Matrigel (Corning Life Sciences, diluted at 1:800) coated 6 well plates (Nunc). Differentiation was started at 80-90% confluency with 6µM CHIR 99021 (Tocris Bioscience) in RPMI B27 minus insulin (Gibco) which was termed day 0. On day 2 CHIR was removed by changing the media to RPMI B27 minus insulin for 24 hours. 2.5µM WNT-C59 (Tocris Bioscience) was added on day 3 and washed out by addition of RPMI-B27 minus insulin on day 4. Cells were maintained in RPMI B27 minus insulin until beating was observed around day 8. Cells underwent metabolic selection with RPMI B27 but without glucose (Gibco) from days 11 to 15, after which the media was changed to RPMI B27 until use. Purity is determined from the percentage of troponin T positive cells in the preparation by histology or FACS analysis: typically this is 95-98% of total (Fig S9).

EHT generation

iPSC-CM were taken 20-30 days post CHIR 99021 treatment and washed twice with Hanks Basic Salt Solution Ca^{2+} free (Thermo Fisher Scientific) and dissociated into single cells for 3.5 hours using dissociation media (Collagenase II 200U/ml (Thermo Fisher Scientific), HEPES 1mM (Thermo Fisher Scientific), BTS 30µM (4-Methyl-*N*-(phenylmethyl)benzenesulfonamide, Tocris Bioscience), Y-27632 10µM (Stem cell technologies) in Hanks Basic Salt Solution).

EHTs were up-scaled to a six well size using the same protocol as the 24 well format (Breckwoldt 2017). The procedure for EHT formation used $20x10^6$ iPSC-CM which were mixed with fibrinogen and thrombin to produce hydrogels: these are suspended from silicone posts and are fed daily until use at four weeks. Cells were collected with RPMI B27 with DNAse 24µg/ml, centrifuged for 15 minutes at 100g. Cells were re-suspended in RPMI B27, counted and separated into tubes of $20x10^6$ cells which made one EHT. Pellets were re-suspended in: 10% Matrigel (Corning), 5.5% 2x DMEM+ (10% horse serum (Gibco), 10% 10xDMEM (Sigma), 1% penicillin/streptomycin (Sigma) in water), 0.1% Y-27632 (10mM), 5mg/ml fibrinogen in non-cardiomyocyte medium (10% fetal bovine serum (Sigma), 1% glutamine (Sigma) in DMEM). The cell suspension was then mixed quickly with 3U/ml thrombin (Sigma) and pipetted into agarose moulds with silicone nets which had been soaked in 5% pluronic for 15 minutes (Sigma). EHTs were left to solidify for 45 minutes, wetted with media and then left a further 15 minutes. EHTs were fed daily with EHT media (10X horse serum (Gibco), 10µg/ml insulin (Sigma), 33µg/ml aprotinin (Sigma), 1% penicllin/streptomycin (Sigma), in DMEM).

To help improve engraftment rates of the hiPSC-CMs, EHTs were subjected to heat shocking and a pro-survival cocktail as previously described immediately prior to implantation (Laflamme et al. 2007). Briefly, EHTs were heat shocked to 43°C for 60 min 24 h before grafting. The pro-survival cocktail consisted of ZVAD (100 mM, benzyloxycarbonyl - Val-Ala-Asp (O-methyl) - fluoromethyl ketone, Calbiochem) Bcl-XL BH4 (cell-permeant TAT peptide, 50 nM, Calbiochem), ciclosporin (200 nM, Wako Pure Chemicals), IGF-1 (100 ng/ml, Peprotech) and pinacidil (50 mM, Sigma). Since the cells were already contained in a hydrogel containing Matrigel, we omitted the step of using Matrigel in the original protocol.

Heart explantation

Animals were anesthetised as described above and intubated but kept at 4% isoflurane for the duration of the procedure. The abdominal aorta and inferior vena cava were exposed via an abdominal laparotomy. With vessels exposed a 18G cannula was placed into the abdominal aorta and the aorta and IVC clamped distally to the cannula insertion site, and then the IVC was cut with slow retrograde infusion of 4°C 300-400ml cardioplegia solution (Harefield Hospital formulation, Terumo BCT) with 12U/ml heparin (Sigma). The heart was carefully freed and transferred in cardioplegia solution with heparin.

Optical Mapping

Optical mapping was carried out at Imperial College London (n=6) and University of Glasgow (n=3). Data were analysed by an investigator blinded to treatment allocation.

Protocol 1

Hearts were mounted in a custom chamber and a volume-conducted pseudoECG was monitored from the chamber. A bipolar platinum stimulating electrode was placed on the epicardial surface at the apical border between the LV/RV, when indicated in the protocol, ventricular pacing was performed using a 1ms square pulse at twice diastolic threshold. Blebbistatin (10 μM) was added to the perfusate to minimise motion artefact during optical recording. Where indicated by the protocol hearts were loaded with a single bolus of 25uL of RH237 (1mg/ml, Molecular Probes, OR) for voltage mapping. Wide-field epicardial optical imaging was performed with a dual CMOS system mounted on a THT macroscope (Micam05, SciMedia, Costa Mesa, CA) with a 20mm^2 field of view resulting in a spatial resolution of ~200μm^2/pixel. All signals were sampled at 1kHz and recordings were made for up to 10s. Hearts were illuminated by LED light sources centred at 475nm (Cairn, UK) and emitted light was collected using a 1x objective lens (Leica, Japan) and split with a dichroic mirror at 662nm. The shorter wavelength moiety, containing the gCaMP6F signal, was bandpass filtered between 575 ±15nm and focussed onto the CMOS sensor. The longer wavelength moiety, containing the RH237 signal (where applicable) was long pass filtered at >715nm. All optical data were processed with a single iteration of a 3x3 Gaussian spatial filter before being analysed using custom software (Optiq, Dr Francis Burton, UoG).

Protocol 2

A 450-470nm LED light source (Cairn, UK) excited the epicardial surface containing the EHT. This was then viewed through a 495nm excitation dichroic to image the calcium transients originating from gCaMP6F hiPSC-CM in the EHT. If spontaneous

signals were not observed the ventricular surface was paced at varying cycle lengths and the EHT was point stimulated with a platinum electrode at 1-2.5Hz. If either a spontaneous or stimulated signal were then observed, hearts then proceeded to undergo a series of ventricular / EHT pacing protocols to ascertain evidence of electrical coupling, by synchronising to the surface ECG (Shadrin et al., 2017). In a subset of hearts, dual mapping of both voltage (Di4ANEPPS at 1 ml of 5 μM) concentration and calcium was used to further assess for electrical coupling. The two-different emission of gCaMP6F and di-4-ANEPPS were separated with a excitation dichroic mirror of 495 nm, and a 610 long pass emission filter (Shadrin et al., 2017).

Assessment of arrhythmia in vivo

Reveal Linq measurements were taken at random intervals on average four times a day for 7.5 min. Indus Telemetry System was set to record continuously at 30 second intervals every five min. All recordings were analysed manually by an experienced cardiologist and any ambiguous traces were checked by a second experienced cardiologist (blinded to group identity). Sustained ventricular was defined as >30 seconds in duration.

Arrhythmia provocation protocol ex vivo

Arrhythmia generation was assessed ex vivo on a Langendorff system by programmed electrical extra stimulus protocols. Pacing was carried out by a MicroPace stimulator with a pacing electrode placed at the base of the left ventricle. Drive train S1 was initiated for 20 beats (cycle length 200ms, 300bpm) followed by adding extra stimuli. If sustained arrhythmia was observed a score is given. If no

arrhythmia was induced burst pacing was initiated at a cycle length between 70-130ms for 50 beats. The susceptibility of each heart to the development of ventricular arrhythmia was categorised using an arrhythmia inducibility score based on the principles as described previously (Belichard et al., 1994). Ventricular tachycardia was defined as non-sustained if >6 but <14 and sustained if >15 were provoked. VF was classified as sustained if >1 second was provoked. Arrhythmias induced on burst pacing were viewed as less arrhythmic than if on extra-stimulus protocols (Table below).

Arrhythmia provocation protocol scoring system	
**Induced arrhythmia**	**Score**
Sustained VT / VF on S1S2	7
Non-sustained VT / VF on S1S2	6
Sustained VT / VF on S1-S3	5
Non-sustained VT / VF on S1-S3	4
Sustained VT / VF on S1-S4	3
Non-sustained VT / VF on S1-S4	2
Any VT / VF on burst pacing protocol	1
No arrhythmia induced	0

Antibody staining

Rabbit hearts were fixed for 24 hours with 4% Histofix (Carl Roth P087.3) and cut into 2-4mm sections before being stored in 70% alcohol solution. Sections were embedded into paraffin and cut into 5µm slices. Slides were dewaxed by Neoclear (Sigma) and antigen retrieval by boiling in pH6 citrate buffer. Immunohistochemical staining was carried out using ABC kit (ThermoFisher 32020) and DAB Kit (Vector Labs, SK-400) using the manufacturers protocol. Immunofluorescence was carried out using standard protocols but using Alexa Fluor 594 and 680 for dual labelling.

EHTs were washed in PBS and fixed in 4% Histofix (Carl Roth) for 24 hours and cut into three pieces with the middle of the EHT taken for analysis.
Blocking/permeabilization was carried out with Tris-buffered saline 0.05M, pH 7.4, 10% FCS, 1% BSA, 0.5% Triton X-100 for 24 hours at 4°C. Primary and secondary antibody incubation was carried out in the same blocking/permeabilization solution minus FCS also for 24 hours at 4°C, with standard 3x5 min PBS washes in-between. Antibodies used are listed in Supplementary Table 1.

Histology

Haematoxylin/Eosin and Pico Sirius staining were carried out by standard methods. Briefly, Harris Haematoxylin was applied for 5-8 minutes to deparaffinised and rehydrated slides. Slides were washed with water then 1% hydrochloric acid / 70% ethanol and stained with Eosin for 2 minutes. Slides were washed with water and dehydrated followed by Neoclear and then mounted. Pico Sirius red staining was carried out for 60 minutes after Harris Haematoxylin staining. Slides are washed in 0.5% Acetic Acid in deionised water twice, followed by dehydration and Neoclear treatment. All slides were mounted in DPX mounting medium.

Analysis of heart sections

Heart sections were analysed using ImageJ. The EHT was outlined and colour deconvolution was applied to separate blue haematoxylin and brown DAB signals. Haematoxylin was used to calculate total EHT area and DAB used for antibody signal. Cell counter plugin was then used to calculate the capillary density by

manually counting capillaries within the EHT. Troponin T positive signal was calculated in a similar way as capillary density but using DAB signal to measure the area of the Troponin T positive cells and haematoxylin for total EHT area. The percentages were then normalised to EHTs which had been placed on the heart and sacrificed at day 0. Analysis of proliferation was carried out automatically using cell profiler. The pipeline consisted of automated thresholding to identify MYH6 positive cells, DAPI positive nuclei and ki67 positive nuclei. Filtering using typical diameter of nuclei was carried out to exclude any identified objects that were not nuclei. All identified objects were then related to each other with nuclei positive for MYH6 counted as cardiomyocytes and the nuclei positive for DAPI, MYH6 and Ki67 counted as proliferating cardiomyocytes.

Contractility measurements

Contraction kinetics for EHT before implantation were taken by mounting a plate on a 3D custom printed apparatus with XYZ movement and videos were recorded using a 100 f.p.s camera (Basler acA720-520µm). Recordings were taken at 0Hz, 1Hz and 2Hz paced electrical stimulation frequencies. A free open source program MUSCLEMOTION was used to detect the movement of EHTs as a plugin into ImageJ where parameters: time to peak, relaxation, and contraction time at 90% were recorded (Sala 2017).

Calcium Transients

EHTs generated with gCamp6f calcium sensitive reporter were analysed at early (4-14 days) or late (>28 days) at 37°C with 5% CO_2. Videos were acquired at 500 f.p.s

for 4 seconds and numbers extracted from changes in fluorescence were analysed in pClamp.

References

Belichard, P., Savard, P., Cardinal, R., Nadeau, R., Gosselin, H., Paradis, P. and Rouleau, J. L. (1994) 'Markedly different effects on ventricular remodelling result in a decrease in inducibility of ventricular arrhythmias', Journal of the American College of Cardiology. doi: 10.1016/0735-1097(94)90440-5.

Brekwoldt, K. *et al*. Differentiation of cardiomyocytes and generation of human engineered heart tissue. Nat. Protoc. (2017). Doi: 10.1038/nprot.2017.033.

Laflamme, M. A., Chen, K. Y., Naumova, A. V., Muskheli, V., Fugate, J. A., Dupras, S. K., Reinecke, H., Xu, C., Hassanipour, M., *et al.* Cardiomyocytes derived from human embryonic stem cells in pro-survival factors enhance function of infarcted rat hearts. *Nat. Biotechnol.* (2007) doi:10.1038/nbt1327.

Sala, L. *et al.* MUSCLEMOTION. A Versatile Open Software Tool to Quantify Cardiomyocyte and Cardiac Muscle Contraction In Vitro and In Vivo. *Circ. Res.* (2017). doi:10.1161/circresaha.117.312067.

Shadrin, I. Y. *et al.* Cardiopatch platform enables maturation and scale-up of human pluripotent stem cell-derived engineered heart tissues. *Nat. Commun.* (2017). doi:10.1038/s41467-017-01946-x

Supplementary Figures

Supplementary Figure S1: Distribution of hiPSC-CM in EHT on control animals

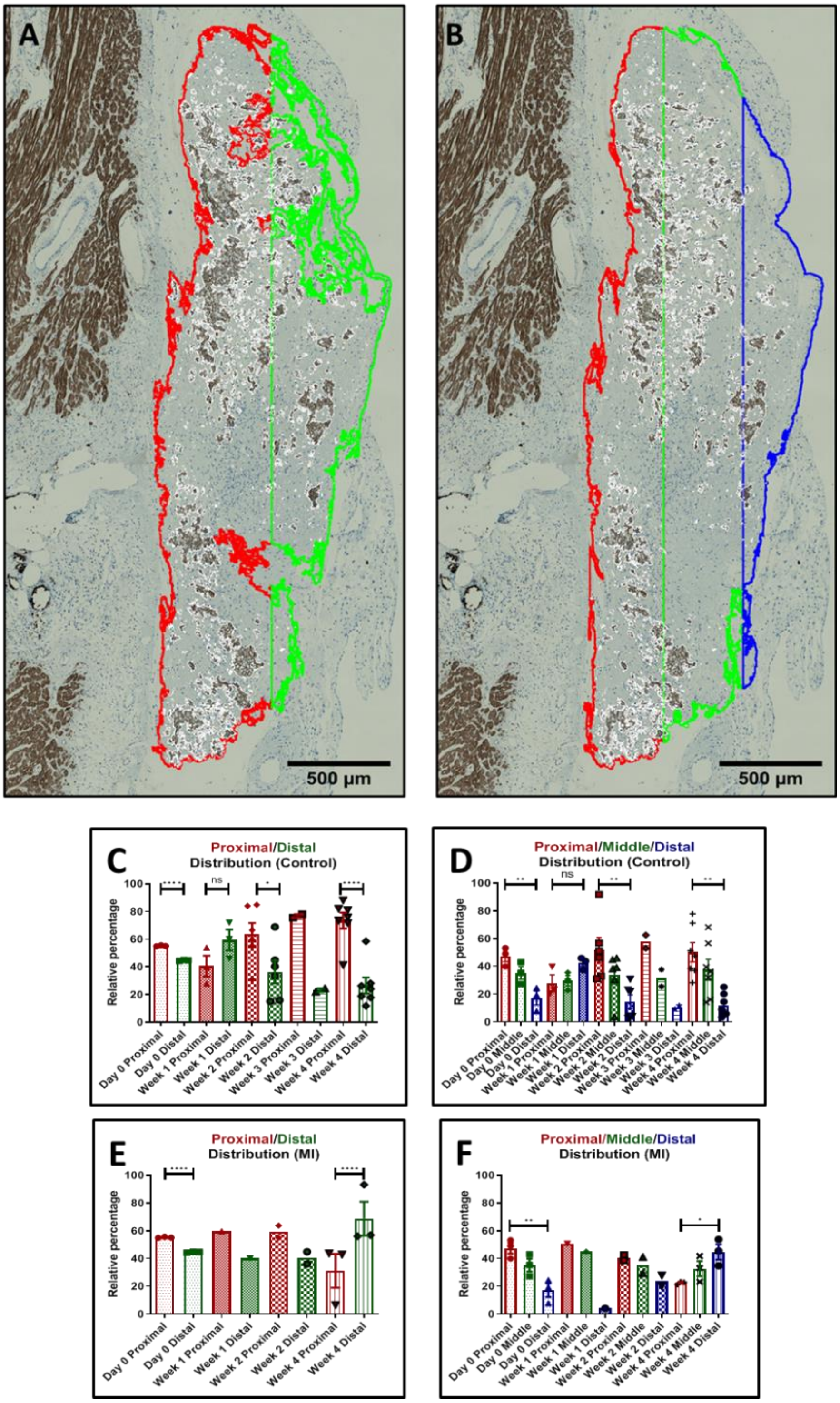

(A) Representative image showing distribution of Troponin T positive cells (brown) in proximal (red) and distal (green) to the host heart and (B) showing distribution in proximal (red), middle (green) and distal (blue) to the host heart. Graphs showing

proximal/distal distribution (C) and proximal/ middle/distal distribution (D) in control hearts (Day 0 n=3, week 1 n=3, week 2 n=6, week 3 n=2 and week 4 n=7) and proximal/distal distribution (E) and proximal/middle/distal distribution (F) in MI hearts (Day 0 n=3, week 1 n=1, week 2 n=2 and week 4 n=3). Scale bar = 500 µm.

Supplementary Figure S2: Ku80 positive nuclei in control group

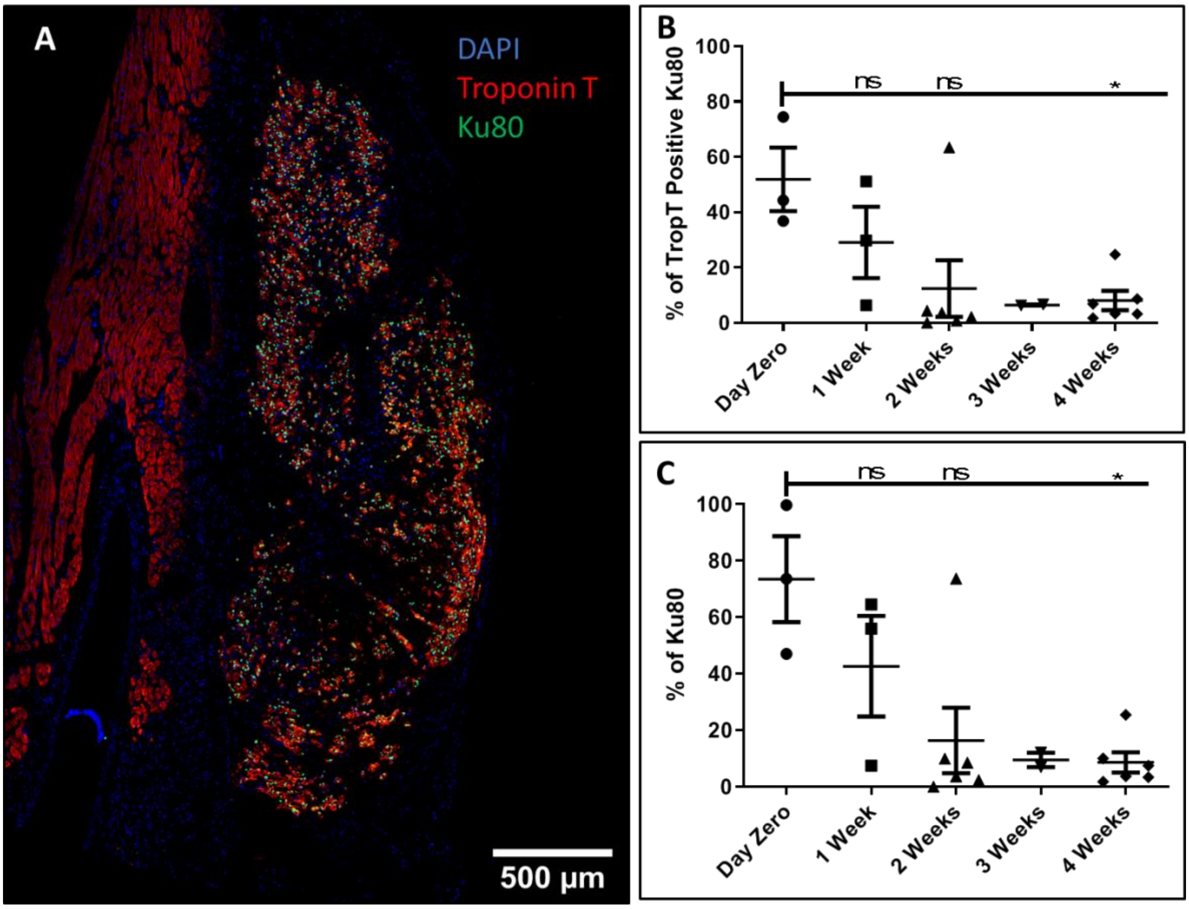

(A) Immunofluorescent labelling of Troponin T positive cells (red), Ku80 positive nuclei (green) and DAPI (blue). Percentage of cells positive for both Troponin T and Ku80 (B) and percentage of all Ku80 positive cells (C) at different timepoints. $P<0.05$. Scale bar = 500 µm.

Supplementary Figure S3: Fibroblast content of engrafted EHT

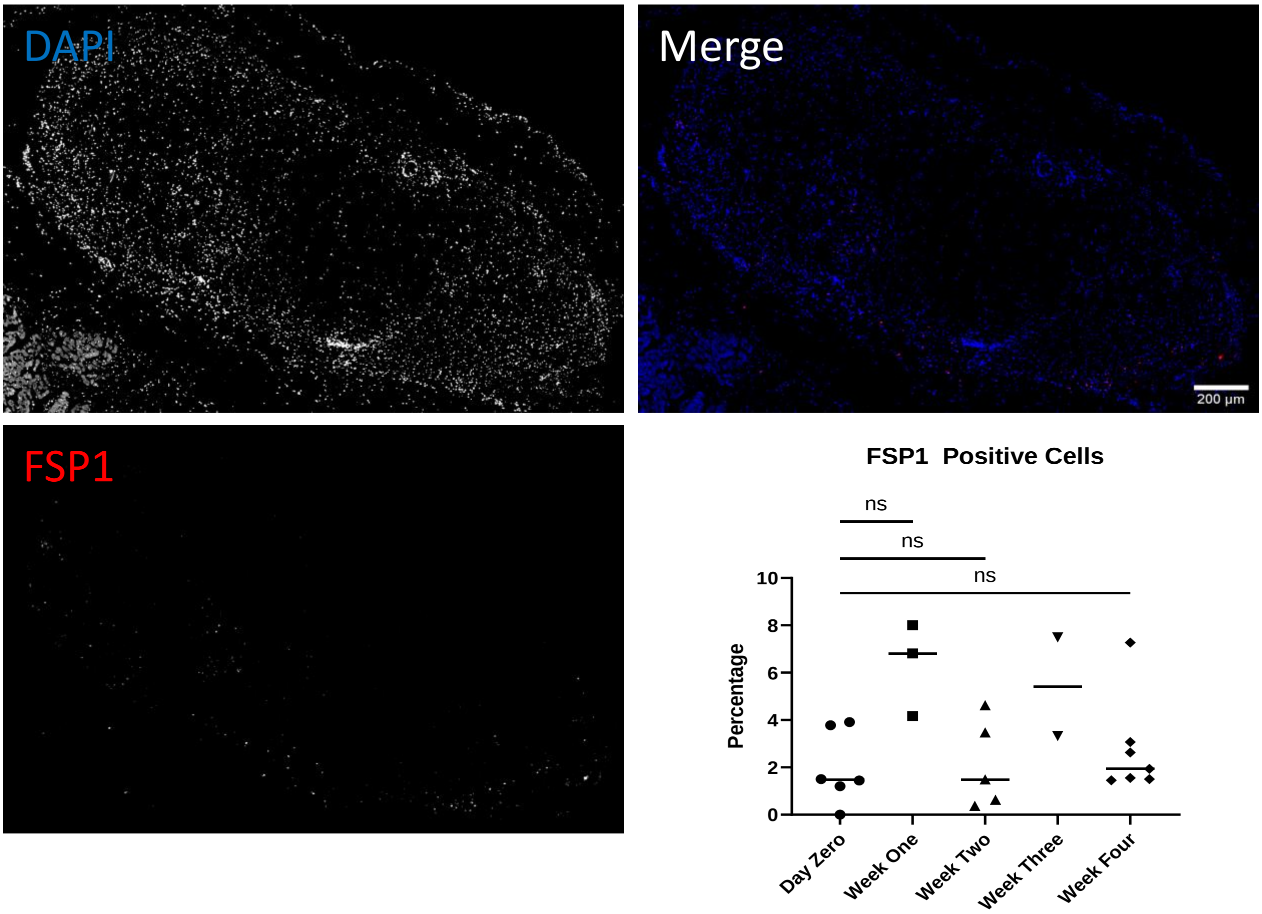

Representative images of cells within EHT positive for fibroblast marker FSP1. Graph shows proportion of cells at each week following implantation, compared with Day 0.

Supplementary Figure S4: Graft Proliferation in control group

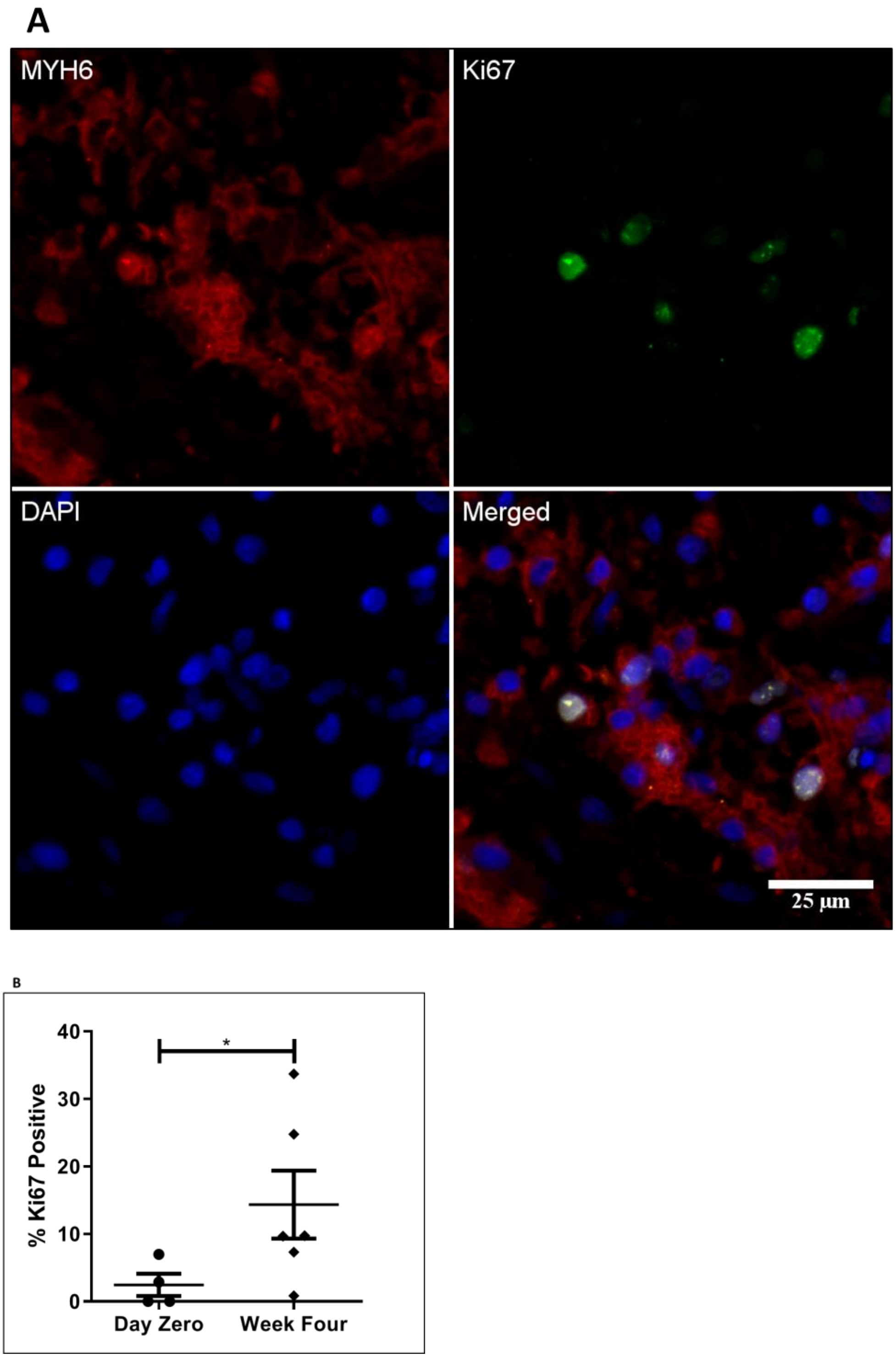

(A) Representative image of myosin heavy chain 6 (red), ki67 (green) and DAPI (blue). (B) Graph showing percentage of ki67 positive cells at day zero and four weeks after EHT transplantation. Scale bar = 25µm, $P<0.05$.

Supplementary Figure S5: Cellular Alignment in control group

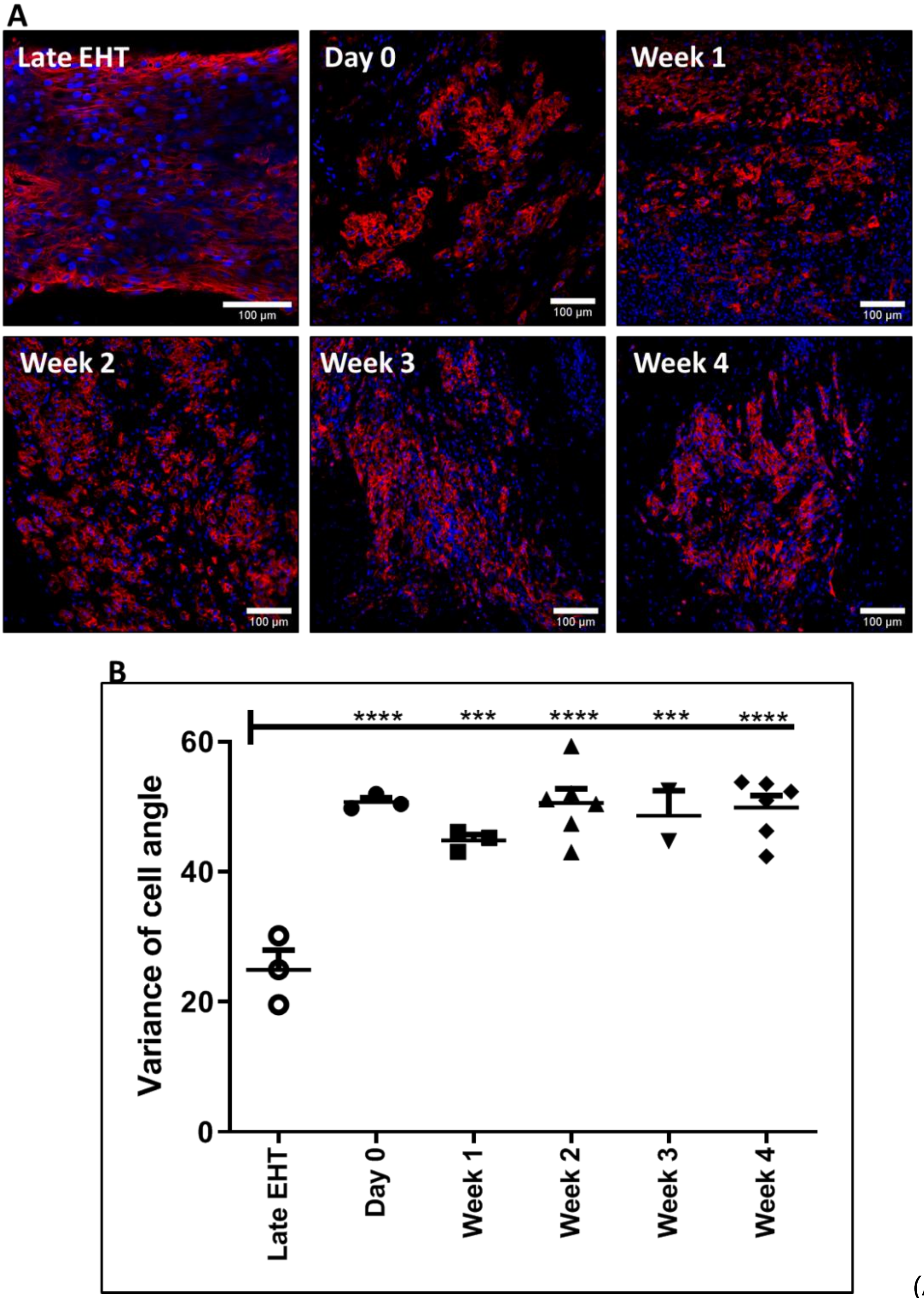

(A)

Alignment of Troponin positive cells (red) in late EHT and at day 0, week 1,2,3 and 4. (B) Graph of variance of cell angle when compared to mean angle of cells at the different timepoints. P < 0.001 - 0.0001. Scale bar = 100 µm.

Supplementary Figure S6: MI retention

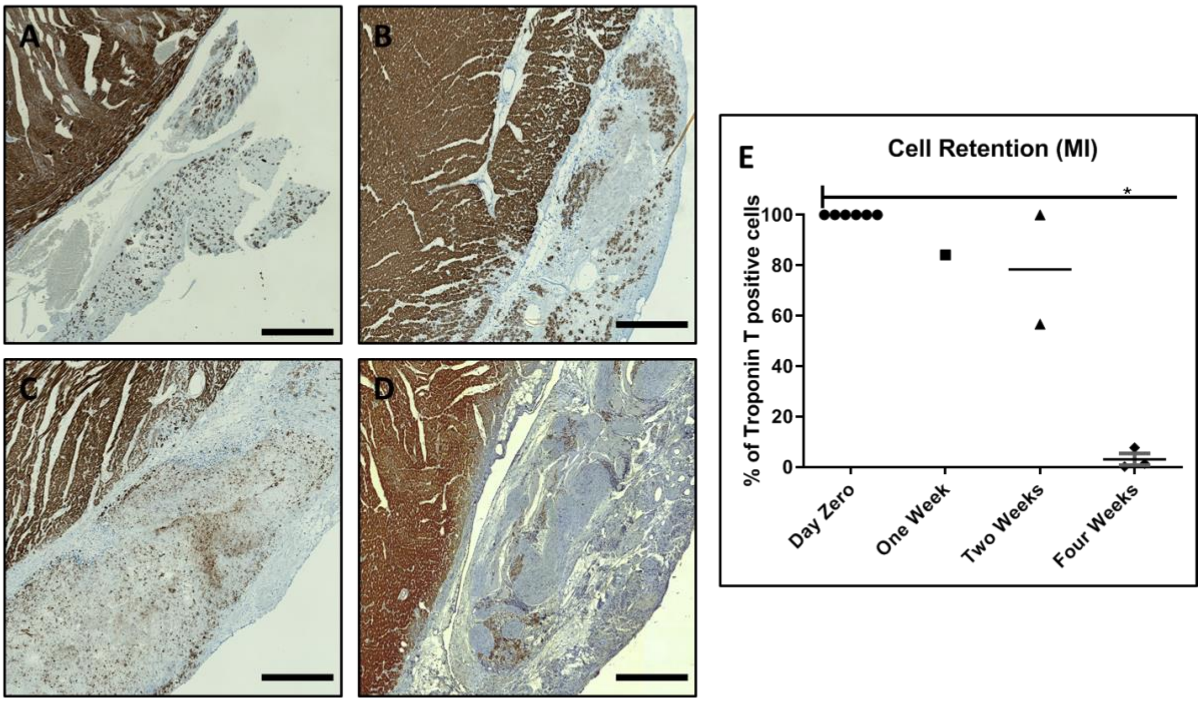

DAB staining of Troponin T positive cells (brown) at day 0 (A), week one (B), week two (C) and week four (D) EHTs grafted onto MI hearts. (E) Graph showing % of troponin T positive cells at the four timepoints. $P < 0.05$. Scale bar = 500 µm.

Supplementary Figure S7: MI vascularisation

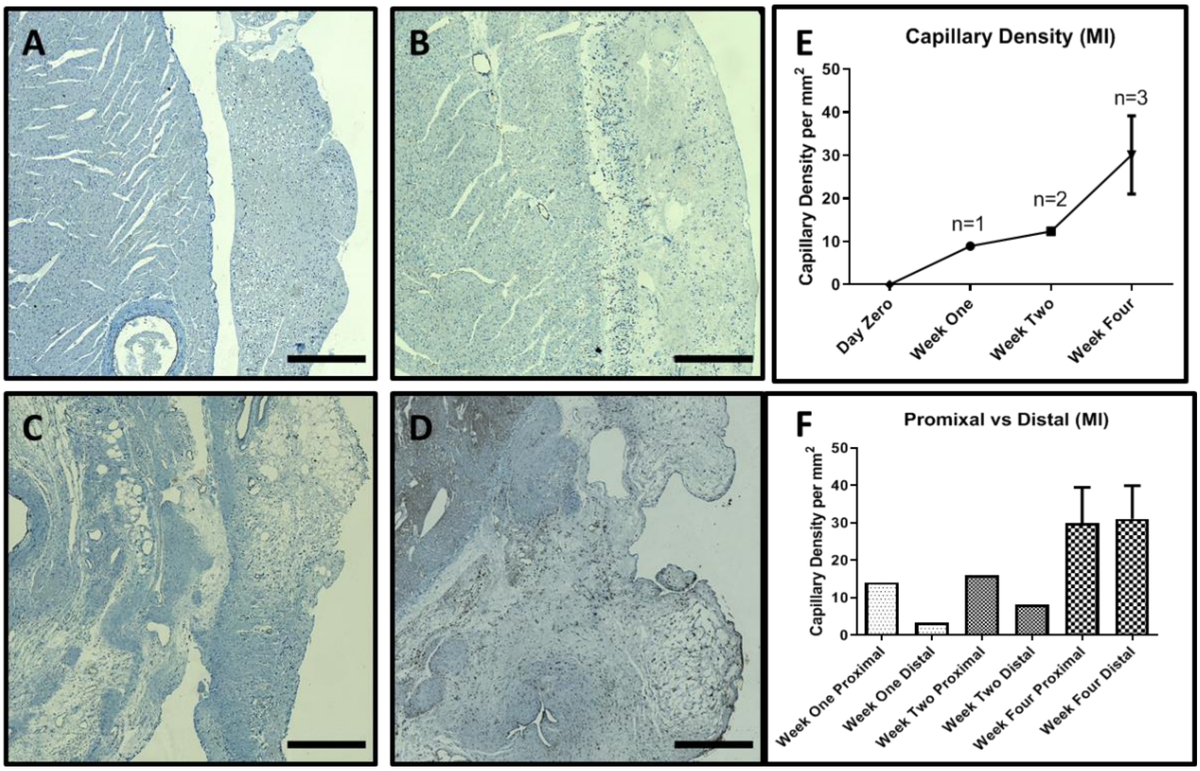

DAB staining of CD31 positive cells (brown) at day zero (A), week one (B), week two (C) and week four (D) in MI hearts. (E) capillary density per mm^2 at four different timepoints and distribution of capillaries at proximal and distal regions to the host heart (F). Scale bar = 500 µm.

Supplementary Figure Supplementary Figure S8: Immune cell infiltration into EHT

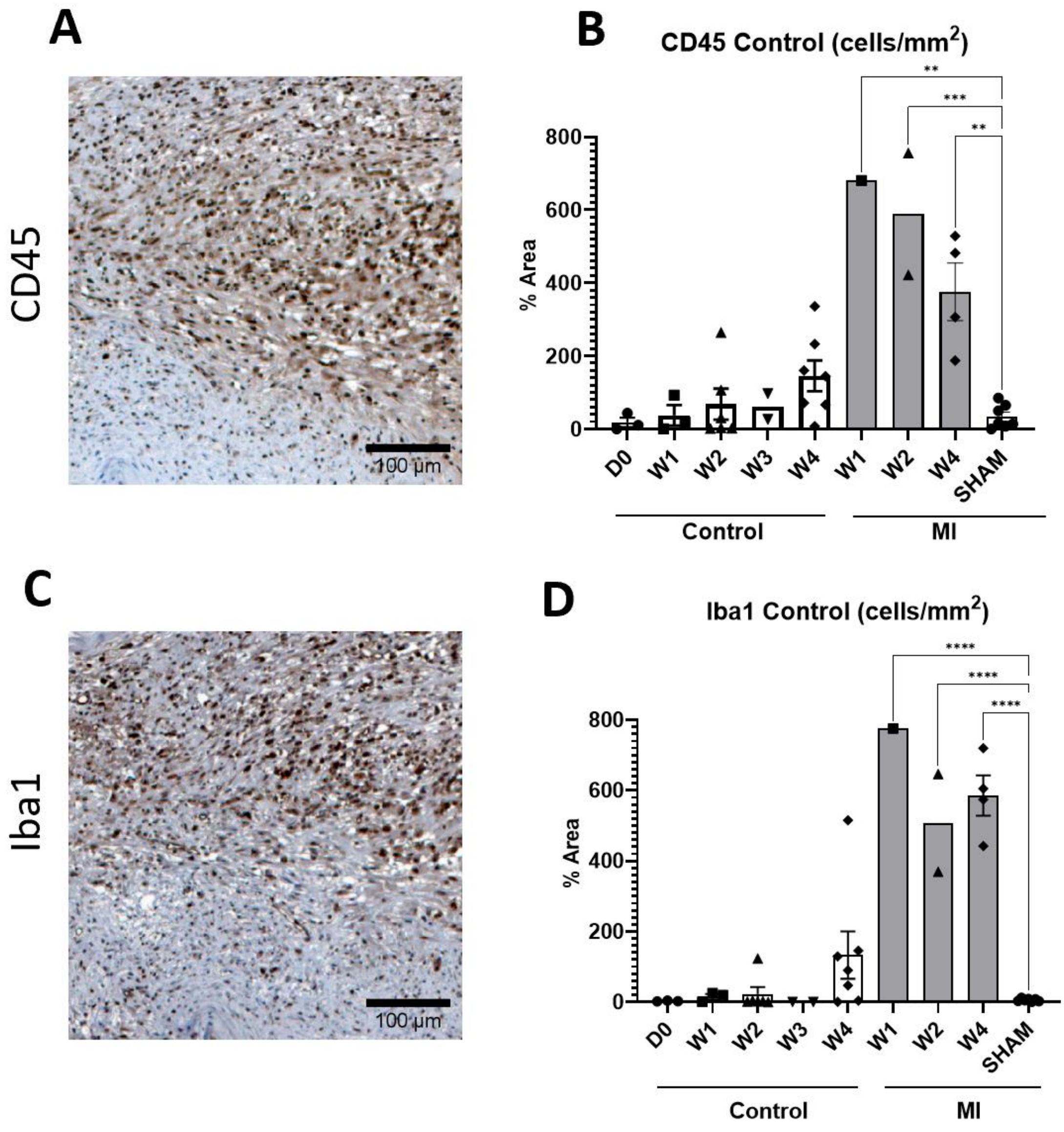

A, C Representative images EHT on explanted hearts (blue haematoxylin staining of all nuclei and brown DAB staining of CD45 and Iba1 positive cells). B,D CD45 and Iba1 positive cells per mm^2 at weeks 1-4 after implantation. P<0.02, P<0.001 vs Day0 (control, non-infarcted) or Sham cell-free patch (MI), ANOVA.

Supplementary Figure S9: Purity of hiPSC-CM

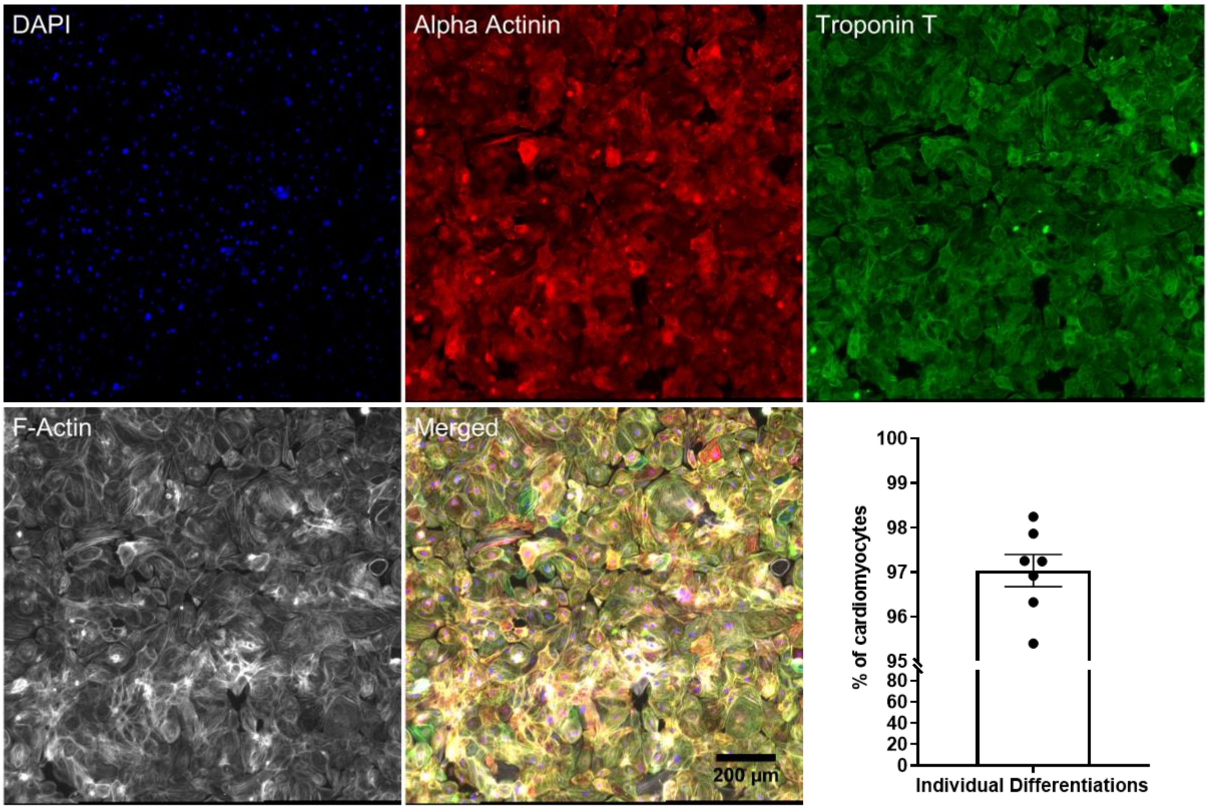

Representative images of differentiated hiPSC-CM at d30 after differentiation, with graph showing percentage of troponin T positive cells in 6 consecutive differentiations.

Supplementary Table S1: Antibodies used in this study.

Antibody	Concentration	Company
Mouse monoclonal to Cardiac Troponin T	4 µg/mL	Abcam (ab10214)
Rabbit polyclonal to Cardiac Troponin T	2 µg/mL	Abcam (ab45932)
Mouse monoclonal to PECAM1	40 µg/mL	Novus Biologicals (NB600-562)
Mouse monoclonal to Ku80	4 µg/mL	Abcam (ab119935)
Rabbit polyclonal to Ki67	1 µg/mL	Abcam (ab15580)
Rabbit polyclonal to CD45	5 µg/mL	Abcam (ab10558)
Goat polyclonal to Iba1	5 µg/mL	Abcam (ab5076)
Mouse Monoclonal [3-48] to MYH6	1 µg/mL	Abcam (ab15)
Biotin Goat Anti-Mouse IgG (H+L)	2 µg/mL	Jackson ImmunoResearch (115-065-003)
Biotin Donkey Anti-Goat IgG (H+L)	2 µg/mL	Abcam (ab6884)
Alexa Fluor 594 Goat Anti-Rabbit IgG	2 µg/mL	Jackson ImmunoResearch (111-585-144)
Alexa Fluor 680 Goat Anti-Mouse IgG	2 µg/mL	Jackson ImmunoResearch (115-625-146)

Supplementary Videos

Supplementary Video 1. Contraction of early EHTs

Supplementary Video 2/3. Contraction of late EHTs

Supplementary Video 4. Late gCamp6f EHTs with 2,3-butanedione monoxime.
